# 160 GHz Schottky Diodes from Solution‐Processed IGZO

**DOI:** 10.1002/smll.202504148

**Published:** 2025-12-29

**Authors:** Lazaros Panagiotidis, Hendrik Faber, Yiyang Yu, Spyridon Doukas, Linqu Luo, Mohammed Ghadiyali, George T. Harrison, Dipti Naphade, Suman Mandal, Wejdan S. Alghamdi, Harold F. Mazo‐Mantilla, Temur Maksudov, George S. Pappas, Udo Schwingenschlögl, Shadi Fatayer, Elefterios Lidorikis, Atif Shamim, Thomas D. Anthopoulos

**Affiliations:** ^1^ Physical Science and Engineering Division KAUST Solar Center (KSC) King Abdullah University of Science and Technology (KAUST) Thuwal Saudi Arabia; ^2^ Computer, Electrical and Mathematical Science and Engineering Division King Abdullah University of Science and Technology (KAUST) Thuwal Saudi Arabia; ^3^ Department of Materials Science and Engineering University of Ioannina Ioannina Greece; ^4^ Henry Royce Institute Photon Science Institute Department of Electrical and Electronic Engineering The University of Manchester Manchester UK

**Keywords:** flash lamp annealing, indium‐gallium‐zinc‐oxide, large area electronics, radio frequency electronics, Schottky diodes

## Abstract

Traditional radio frequency (RF) electronics rely on discrete devices, such as diodes, transistors, capacitors, and antennas capable of operating in the GHz frequency domain. Unfortunately, integrating these components to realize large‐area RF electronics presents formidable challenges. Herein, we demonstrate wafer‐scale, solution‐processed indium‐gallium‐zinc‐oxide (IGZO) Schottky diodes with a cut‐off frequency exceeding 160 GHz. The diodes feature planar asymmetric self‐aligned nanogap electrodes patterned via adhesion lithography (a‐Lith) and a flash lamp annealed solution‐processed IGZO as the semiconducting layer. An enabling feature of the devices is the Ohmic contact facilitated by an ultra‐thin (10 nm) ZnO layer deposited atop the aluminum electrode without increasing the manufacturing complexity. The ZnO interlayer shifts the work function of the aluminum electrode closer to the conduction band of IGZO, reducing the injection barrier and improving electron injection. The ensuing diodes show reduced turn‐on voltage (≈0.08 V), higher on‐current, high rectification ratio (≈10^5^), and ultra‐low junction capacitance (<15 pF). RF rectifier circuits made of these IGZO diodes yield a maximum output DC voltage of 0.74 V with an extrinsic cut‐off frequency exceeding 160 GHz, making them the fastest large‐area diodes reported to date. Our technology offers scalable manufacturing with unprecedented performance and creates new opportunities for emerging applications.

## Graphical abstract

A simple, scalable method for contact engineering of planar asymmetric nanogap electrodes enables the development of Schottky diodes with a cut‐off frequency of 160 GHz. Applying these diodes to radio‐frequency rectifying circuits yields output voltages in the range of 0.3–0.74 volts.

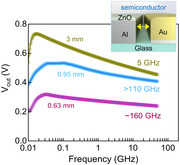

## 1 Introduction

The rapid evolution of wireless telecommunication, computing, storage, and sensors has opened the path to establish and commercialize highly interconnected networks such as the Internet of Things (IoT) [1, 2]. With 5G networks already a commercial reality research is now focusing on the next generation (6G) technologies, able to operate beyond 95 GHz [3, 4]^.^ Meanwhile, the advancements and increasing demand in the area of artificial intelligence (AI) and related applications necessitate the implementation of networks able to operate in the millimeter (30–300 GHz) and terahertz (0.3–10 THz) wave‐bands [4, 5, 6]. Losses at these wavelengths due to indoor penetration, signal attenuation, and limited propagation capability pose formidable challenges for such technologies [7]. Reconfigurable intelligent surfaces (RISs) offer a potential solution to these challenges by enabling phase‐shift surfaces that create a propagation channel between the transmitter and the receiver [8, 9, 10]. Practical implementation of RISs, however, relies on RF components able to operate at this frequency domain and are manufactured over large‐area surfaces economically [3, 11, 12].

Among the various RF components, Schottky diodes are considered a fundamental component of rectifier circuits, mixers and energy harvesting systems due to their high cut‐off frequency, low turn‐on voltage and good current‐driving capabilities [12, 13, 14]. By engineering the Ohmic contact and the Schottky barrier at the metal‐semiconductor‐metal junctions, high output voltage, high on‐current and high operating frequency can be achieved [15, 16]. Most commercial Schottky diodes are based on silicon (Si). However, high annealing temperatures (often >1000°C) significantly increase the fabrication cost while posing severe limitations in deploying these devices in emerging applications such as large area electronics. To address these challenges recent research is focusing on III‐V semiconductors, two‐dimensional materials (2D) and metal oxides [17, 18, 19, 20]. III‐V semiconductors offer higher carrier mobility, direct bandgap, and smaller electron effective mass, rendering them attractive for a plethora of applications [21]. 2D materials on the other hand offer high carrier mobility and tunability in their optoelectronic properties, making them attractive candidates for integrated circuits (ICs) [22, 23, 24]. However, the fabrication methods used in both cases present significant limitations such as high‐temperature processing, high manufacturing cost and incompatibility with inexpensive large area substrates [25].

Metal oxide (MOx) semiconductors could address a key limitation in terms of scalable processing while simultaneously offering attractive properties such as high carrier mobility and optical transparency [13, 26, 27, 28, 29, 30, 31, 32]. However, a critical challenge of MOx devices is achieving the required performance metrics without compromising manufacturing simplicity. For example, to develop Schottky diodes with a high cut‐off frequency (*f*_C_), their junction capacitance (C_j_) and the series resistance (R_s_) need to be minimized without increasingly complex fabrication protocols. For example, vertical diodes are simpler to make but often exhibit high capacitance [33], which can be mitigated by reducing the device dimensions but at the cost of current driving capabilities. Planar architectures provide an alternative solution due to their ultra‐low capacitance (∼fF) [34, 35, 36]. Unfortunately, high‐frequency planar devices rely on complex manufacturing protocols and/or difficult‐to‐scale semiconductors, hindering their deployment in large‐area RF electronics. The use of alternative patterning paradigms for the scalable manufacturing of planar devices has also been proposed with some success [11, 37, 38, 39]. Despite the increasing efforts, the reduction of critical device dimensions without compromising the overall processability remains a key challenge.

Adhesion lithography (a‐Lith) is a promising method that enables scalable patterning of planar asymmetric metal electrodes that are spaced only a few tens of nanometers apart [13, 40]. Because of these unique capabilities, a‐Lith was recently used to develop metal oxide and organic Schottky diodes with record cut‐off frequencies [13, 14]. Loganathan et al. advanced the method further and demonstrated IGZO diodes with an extrinsic cut‐off frequency (*f*_ext_) of at 47 GHz [3]. Despite the unprecedented *f*_ext_, the diodes exhibited significant contact resistances and, when integrated into rectifier circuits, limited the DC voltage output to ≈ 0.3 V. Exploring a similar approach, Yang et al. [41] combined a planar device architecture and contact engineering with 2D MoS_2_ semiconductors to demonstrate logic circuits. Although limited, these preliminary studies highlight the technological potential of the planar diode architectures over traditional vertical devices.

Here we report the development of photonically processed IGZO Schottky diodes with the highest extrinsic cut‐off frequency reported to date. We achieve this through concurrent advancements in electrode nanopatterning and charge injecting contact engineering without increasing the overall manufacturing complexity. The ensuing n‐type IGZO Schottky diodes combine impressively low junction capacitance (< 15 pF) with low series resistance leading to a remarkable cut‐off frequency of over 160 GHz. The diode's extraordinary manufacturability and record cutoff frequency creates new opportunities for future applications in emerging areas such as rectennas for wireless RF energy harvesting and reconfigurable intelligent surfaces for 6G communications.

## 2 Results and Discussion

**FIGURE 1:**
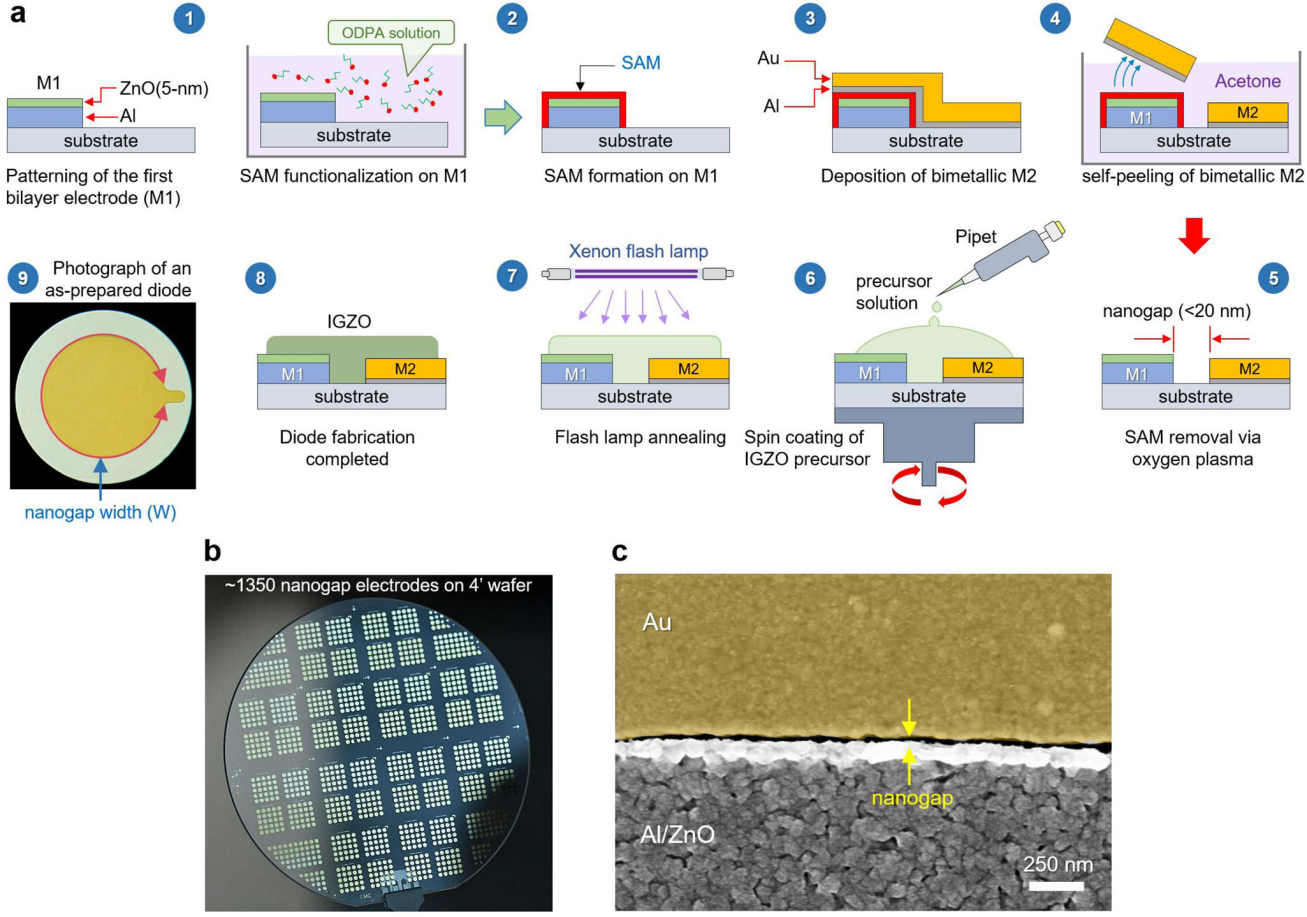
Fabrication of contact engineered nanogap Schottky diodes. a) Schematic representation of the adhesion lithography process steps. The essential steps include the patterning and the functionalization of the SAM on the Al/ZnO (M1) electrodes (1‐2), followed by the Al/Au (M2) deposition (3), leading to the selective self‐peeling of M2 in a heated acetone bath, resulting in empty planar metallic contacts separated by a nanogap (4, 5). The final devices were fabricated by spin coating of the IGZO precursor, followed by a flash lamp annealing (FLA) step (6‐7). An optical microscopy image of a coplanar as‐prepared diode with a width of 3 mm (9). b) A photograph of ∼1350 devices on a 4‐inch wafer. c) False colored SEM image of a continuous and uniform nanogap between M1 and M2.

Planar asymmetric nanogap electrodes comprised of a bilayer aluminum/zinc oxide (metal electrode 1, M1) and gold (metal electrode 2, M2) were fabricated via a‐Lith [40]. The process steps are shown in Figure 1a and described in detail in the Experimental Section. In brief, the poor adhesion properties of M2 on the M1/self‐assembled monolayer (SAM) interface lead to its selective removal via delamination leading to a nanogap formation between the two metallic electrodes. The Octadecylphosphonic acid (ODPA) SAM was used throughout this study as the adhesion force modifier. The removal of M2 via self‐peeling from the surface of M1/SAM is achieved via solvothermal treatment of the unpatterned electrodes by immersing the wafers in an acetone bath for 90 min at 90°C. Any M2 residues are removed from the wafer's surface using mild sonication in an acetone bath. This modified process yields better quality nanogaps that are more homogenous and reproducible compared to previous reports [14]. We attribute this to the elimination of the manual peel‐off step and its replacement with the better‐controlled solvothermal‐based peel‐off step. The latter suppresses the user‐induced variability as well as the risk of contaminating the nanogap region with glue residues, while ensuring complete self‐peeling of the second metal.

For the purpose of this study, a total of ∼1350 circular asymmetric electrode structures were fabricated on each 4‐inch wafer. Figure 1b shows a photograph of the finished wafer an individual concentric circular electrode pairs. For this particular architecture, the circumference of the inner Au electrode defines the width of the nanogap channel. The specific geometric considerations of the diodes are depicted in Figure S1. The channel length (*L*), which refers to the interelectrode distance, was measured using scanning electron microscopy (SEM) (Figure 1c; Figure S2a), atomic force microscopy (AFM) (Figure S2b) and cross‐sectional transmission electron microscopy (TEM) (Figure S2c). All methods consistently show a nanogap length average of 20 nm, with the deviations mainly attributed to the edge roughness and the grain structure of the metal electrodes.

Schottky diodes using the nanogap electrode were developed using IGZO as the n‐type semiconductor deposited by spin‐coating of the precursor solution followed by a photonic sintering treatment. Loganathan et al. [3] showed that planar Al/IGZO/Pt diodes fabricated via the same process exhibit good diode behavior with promising RF performance. However, in the latter work, the formation of an insulating native aluminum oxide (Al_2_O_3_) formed on the surface of the aluminum electrode during processing hindered electron injection and transport across the device, an effect that becomes dominant at these planar nanoscale‐channel devices. Here, we suppressed the formation of Al_2_O_3_ by incorporating a thin zinc oxide (ZnO) as an electron‐injecting interlayer deposited sequentially atop the Al immediately after its deposition. Density Functional Theory (DFT) calculations (Figure S3) reveal that octadecylphosphonic acid (ODPA) binds strongly on both Al_2_O_3_ and ZnO and forms dense SAMs. The sequentially deposited Al and ZnO under an inert atmosphere is expected to suppress the formation of native Al_2_O_3_ while improving the band alignment between the electron‐injecting Al/ZnO electrode and IGZO. Crucially, the DFT calculations (Figure S4a,b) indicate that the density of states (DOS) for the ZnO/IGZO interface exhibits superior charge transfer characteristics compared to the Al_2_O_3_/IGZO interface. This is a significant finding, demonstrating the advantageous properties of the formed ZnO/IGZO interface. Identifying a suitable metal oxide that simultaneously ensures both strong ODPA binding and favorable electronic coupling with IGZO is inherently challenging, as most oxides either form weak phosphonic bonds or create unfavorable energy barriers. ZnO, however, offers the unique combination of chemical compatibility with ODPA and electronic alignment with IGZO, making it an excellent interfacial layer for such device applications.

**FIGURE 2:**
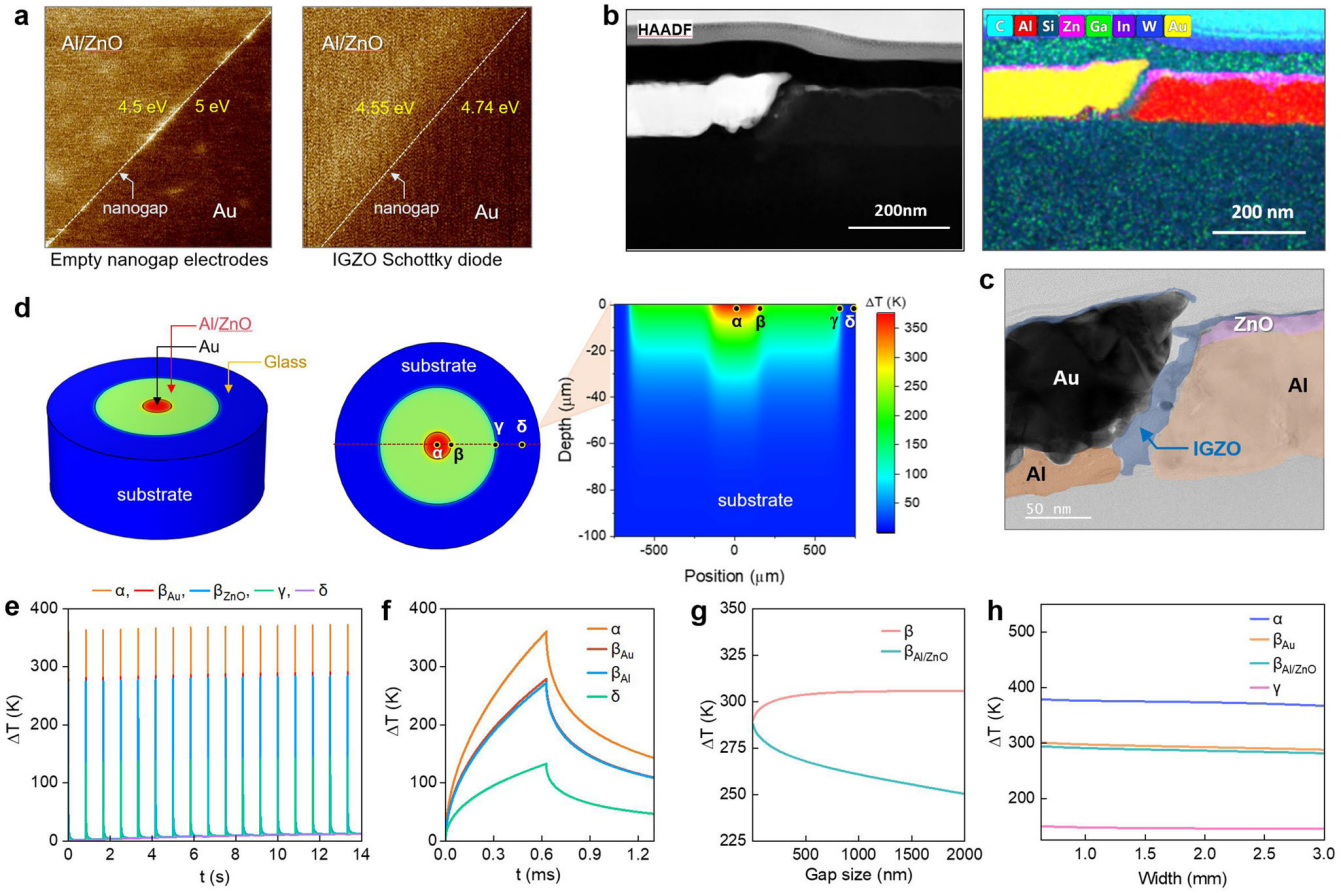
Co‐planar Al/ZnO‐IGZO‐Al/Au diodes via flash lamp annealing (FLA). a) Kelvin Probe Force Microscopy (KPFM) of the nanogap contacts with scan size 15 µm x 15 µm before and after the IGZO deposition to elucidate the work function alteration. b) High resolution STEM images and elemental mapping via Electron Energy Loss Spectroscopy (EELS) revealing In, Ga, Zn and O within the nanogap and c) high resolution TEM (HR‐TEM) image of the cross‐section of the Al/ZnO‐IGZO‐Al/Au nanogap diode after the rapid photonic curing. d) Front (left) and top (middle) 3D representation of a device with W= 0.95 mm and 2D cross‐section (right) of the temperature distribution in three distinct areas of the electrodes (α) Al/Au, (β) Al/ZnO‐Al/Au edge, (γ) Al/ZnO‐Borofloat glass edge and (δ) Borofloat glass. e) Peak temperature rise (ΔΤ) during a full sequence of 20 applied pulses delivering energy of 6 J/cm^2^, and f) time‐dependent temperature rise in the points of interest α, β, γ, and δ during the first pulse. g) ΔΤ at each side of the nanogap, suggesting uniform distribution for gap size < 100 nm. h) ΔΤ evolution in the points of interest (excluding the glass substrate) as a function of the channel width.

Figure 2a displays Kelvin Probe atomic force microscopy (KP‐AFM) images of planar nanogap electrodes comprised of Al/ZnO and Au electrodes before and after IGZO deposition. The Al/ZnO exhibits a work function of 4.5 eV and serves as an Ohmic contact, while the Au metal forms a Schottky contact with IGZO due to its deep work function (5 eV). Following the IGZO deposition, the work functions of both electrodes change with the ohmic Al/ZnO contact, showing a drop of 0.05 eV due to band alignment. AFM topography measurements performed on the contacts before and after IGZO deposition (Figure S5) reveal that electrodes with the semiconductor atop exhibit a smoother surface (i.e. lower root‐mean‐square (RMS) roughness) due to planarization. This observation confirms that the sputtered ZnO interlayer effectively levels local surface irregularities of the underlying Al, resulting in more uniform nanogap interfaces and, consequently, higher fabrication yield. Figure 2b,c and Figures S6 and S7 of the Supporting Information show the cross‐sectional TEM measurements and the corresponding elemental mapping obtained via energy‐dispersive X‐ray spectroscopy (EDX). The results reveal that IGZO fills the nanogap and covers both contacts.

Since complete conversion of the semiconductor precursor to IGZO requires high annealing temperatures (300°C–400°C) [42, 43, 44], opto‐thermal simulations were utilized to determine the temperature transients developed during FLA and their correlation to this process (see Experimental Section and Section ST 5). Figure 2d depicts the temperature distribution at its peak rise during FLA, highlighting four distinct regions of interest: (α) the Au contact, (β) the metal edges of the nanogap, (γ) the Al/ZnO contact, and (δ) the borofloat glass substrate. As shown in Figure 2e, subjecting the device to 20 repeated pulses is sufficient to reach the required temperature and convert the precursor into IGZO. During FLA, heating occurs due to light absorption at the metal contacts, resulting in distinct temperature profiles for each region of interest (Figure 2f). Given the higher absorptivity of the inner Au electrode (Figure S8 and Table S1), the temperature rise (ΔΤ) on its surface is higher (∼375K) compared to the outer Al/ZnO electrode (∼300K). However, due to the proximity of the two electrodes, the temperature distribution within the nanogap remains uniform for gap sizes up to ∼100 nm (Figure 2g). As illustrated in Figure 2h, additional calculations for different channel widths (W) indicate that the temperature increase within the nanogap remains stable, even in devices with varying dimensions. Overall, the simulations demonstrate a uniform temperature rise distribution within the nanogap, with a peak ΔT of ∼300K above ambient temperature. Both the magnitude and uniformity of this ΔΤ demonstrate the suitability of FLA for the rapid conversion of the metal oxide precursor across the nanogap. Crucially, the total processing time remains short, typically less than 16 s.

**FIGURE 3:**
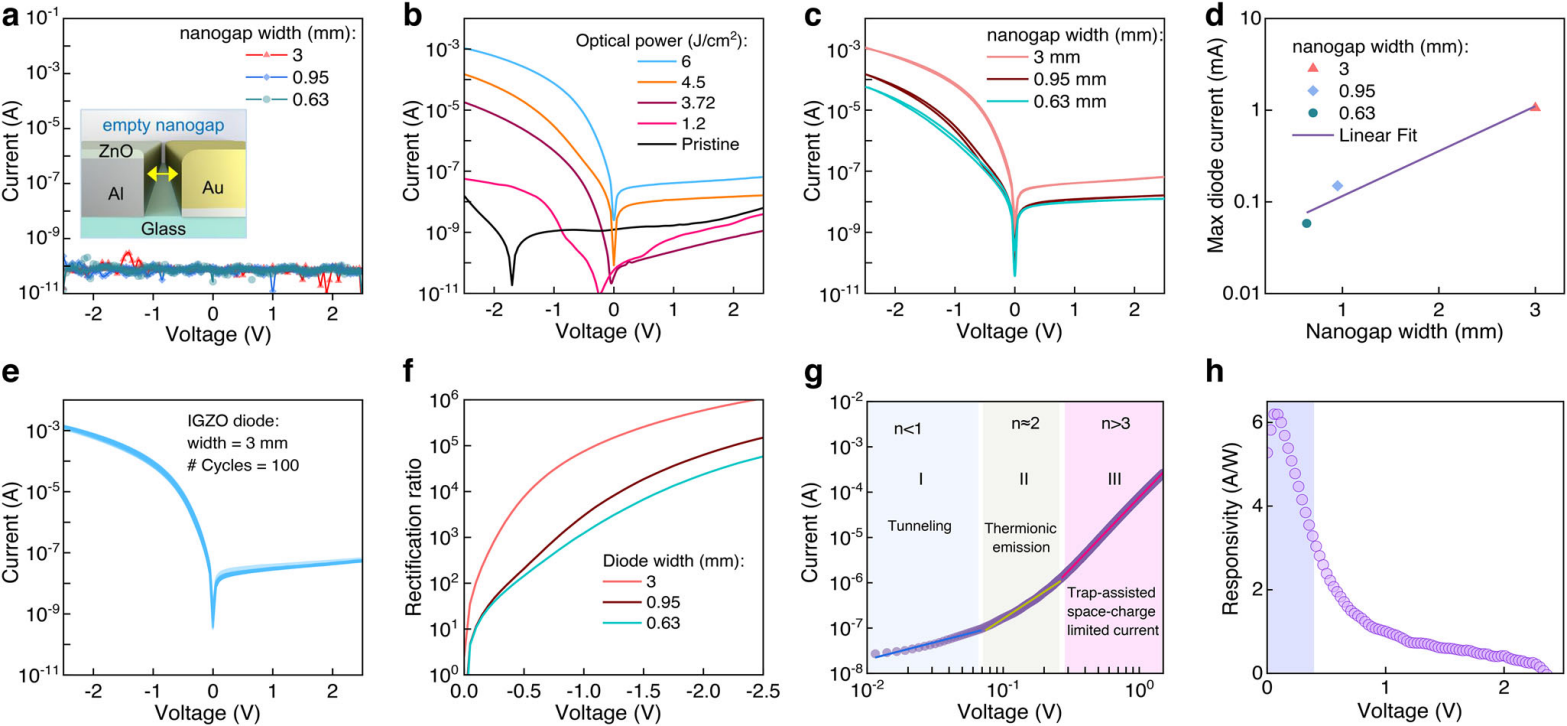
DC measurements and extracted figures of merit of the Schottky diodes. a) *I–V* characteristics of the empty nanogap electrodes, resulting in complete electrical insulation between the two contacts. Inset: 3D representation of the separated nanogap electrodes b) I‐V characteristics of the IGZO diodes processed with different pulse energies. c) I‐V characteristics of the IGZO diodes with different channel widths and d) maximum current of the diodes at −2.5 V, indicating a trend of scaling correlated to the increase of the width. e) Stability test of a 3 mm diode at 100 cycles at a bias range from −2.5 to 2.5 V. f) Rectification ratio for devices with different width W and g) *I–V* characteristics shown in log(I) vs log(V), revealing the three distinct transport regimes corresponding to tunneling (I), thermionic (II) and space‐charge‐limited current (III). h) the quasi‐DC current responsivity of 3 mm width diodes

The most direct method to assess the successful gap formation between Al/ZnO and Au is via current‐voltage (*I–V*) measurements (Figure 3a). As expected, empty nanogap electrodes, i.e. prior to the IGZO deposition, exhibit open circuit characteristics with a low current (∼10^−10^ A) that is independent of the electrodes’ size. The *I–V* characteristics after IGZO has been deposited and treated at varying FLA energy densities are displayed in Figure 3b. In contrast to devices with a pristine IGZO film, which exhibit poor electrical conductivity, the FLA‐treated IGZO devices exhibit n‐type behavior and high *I–V* asymmetry. In addition, there is a steady improvement in device characteristics with increasing FLA power up to the maximum energy density of 6 J/cm^2^, giving rise to a sharper turn‐on and increased forward currents, indicating a successful precursor conversion.

Three different nanogap electrode widths (0.63, 0.95, and 3 mm) were developed to study the geometrical impact on the diodes’ performance (Figure 3c). A clear scaling of the current measured at −2.5 V for diodes with increasing widths is observed (Figure 3d), highlighting the possibility of scaling the device's size to achieve the required current and on/off characteristics. To assess the electrical stability of these devices, a randomly selected 3 mm diode was subjected to 100 repeated *I–V* measuring cycles in the bias range ±2.5 V (Figure 3e). The device exhibits good stability, with no noticeable hysteresis, turn‐on voltage shift, or current degradation. Moreover, the Schottky diodes exhibit high rectification ratios with the larger width diodes reaching ∼10^5^ (Figure 3f), while the smallest diodes (W = 0.63 mm) also demonstrate robust performance with rectification ratios exceeding ∼10⁴.

To further support the structural and electrical performance of our devices after FLA, 55 representative IGZO diodes collected from four different wafers, covering various wafer locations and the three device widths, were tested as illustrated in Figure S9. Among these devices, 52 exhibited clear Schottky diode behavior, corresponding to a 94.5 % operating yield. Notably, 2 of the 3 non‐functional devices while the third was part of the 0.95 mm group. These differences are attributed to local variations in IGZO film formation resulting from non‐uniform energy distribution during the flash‐lamp annealing process, as the system used is not specifically designed for this application. The shorted‐like device characteristics most likely originate from defects introduced during the nanogap electrode fabrication. These effects are attributed to process‐related issues and are not intrinsic to the proposed methodology and/or device architecture. We are confident that the device‐to‐device variability can be drastically improved through process optimization and further automation of each processing step.

The ensuing diodes show ultra‐low junction capacitance of <15 pF, a characteristic attributed to their nanogap coplanar architecture (Figure S10). Further analysis of the forward bias current in Figure 3g reveals three distinct transport regimes, namely, (i) tunneling, (ii) thermionic emission and (iii) space charge limited conduction (SCLC), resembling conventional Schottky diodes despite the planar architecture. Using capacitance‐voltage (C–V) and *I–V* analysis (Figures S10 and S11), crucial Schottky junction parameters, including barrier height (ΦΒ), series resistance (Rs), built‐in potential (Vbi), ideality factor (n) and carrier concentration (ND), were also extracted (Table S2). A key figure of merit for any Schottky diode intended for radio‐frequency (RF) energy harvesting applications is its current responsivity. For example, it is known that high power conversion efficiency is achieved when at low applied bias, high responsivity values (>6 A/W) can be achieved [3, 13, 45]. In Figure 3h we calculated the low‐level quasistatic DC responsivity of a representative 3 mm IGZO diode, yielding 6.2 A/W at 0.07 V. This remarkable characteristic highlights the huge potential of our planar diodes for RF applications.

**FIGURE 4:**
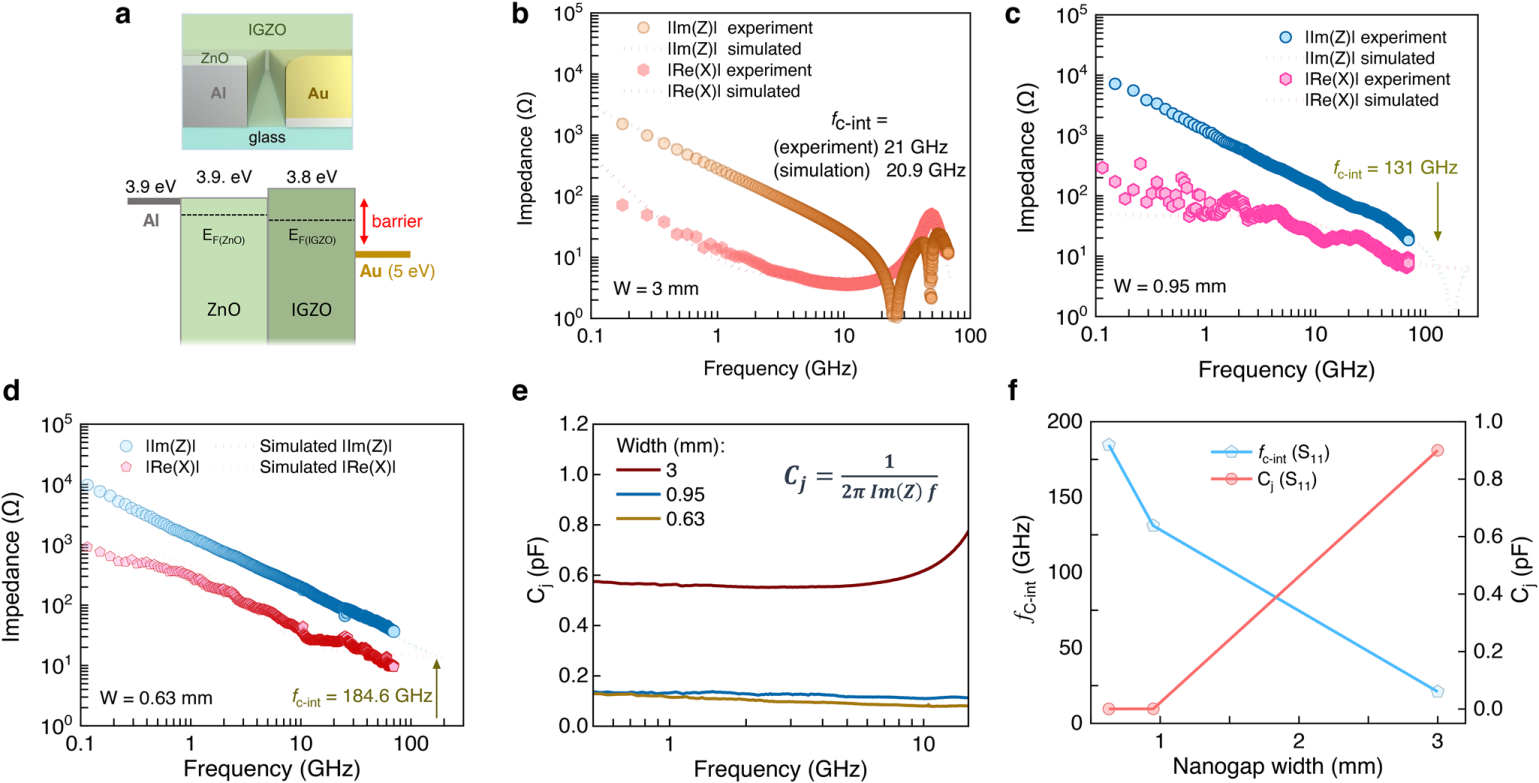
Energy levels and high‐frequency measurements (S_11_ parameters) of the IGZO diodes. a) 3D representation of the coplanar Schottky diode and the energy band diagram of Al/ZnO‐IGZO‐Al/Au constructed based on UPS (Ultraviolet Photoelectron Spectroscopy) (Figure S17) and Kelvin probe measurements (dashed lines represent the work function of IGZO and ZnO). b–d) Experimental and simulated frequency‐dependent impedance of the IGZO diodes derived from the S_11_ measurements, varying with channel widths. e) Junction capacitance (C_j_) of the diodes based on the S_11_ measurements, ranging from 0.5 GHz to 15 GHz, with recorded values below 1 pF. f) Intrinsic cut‐off frequency and junction capacitance of the IGZO diodes of different widths, extracted from the S_11_ measurements.

Owing to the bilayer nature of the electron‐injecting Al/ZnO contact architecture, the ensuing devices combine a high on‐current with lower junction capacitance and a notably lower series resistance compared to our previous studies [3, 12, 13, 14, 29]. The use of the ZnO interlayer is essential for two main reasons. First, the spontaneous formation of the insulating native Al_2_O_3_ on the Al contact is prevented by the presence of ZnO. Second, ZnO shifts the work function of the electron‐injecting Al contact by improving the band alignment at the Al/IGZO interface. Specifically, in Figure 4a, the Fermi energy difference between ZnO and IGZO is only ∼0.1 eV. This favorable energy alignment facilitates efficient electron injection, while on the other side of the device, the Au/IGZO interface provides a large Schottky barrier for electrons moving from the Au side to the conduction band of IGZO. These concurrent improvements enhance diode performance, underscoring the bilayer Al/ZnO contact architecture as an effective solution to the limitations encountered in our earlier works [3, 13, 14].

The frequency response of the planar Al/ZnO/IGZO/Au diodes was studied through one‐port scattering parameter (*S_11_*) measurements (Figures S12 and S13). The reflection of high‐frequency input signals from 10 MHz to 67 GHz was experimentally determined, and the capacitive and inductive behavior of our devices was extracted. The intrinsic cut‐off frequency (*f*_c‐int_) is estimated from the intersection point of the real (resistance) and imaginary (reactance) parts of the impedance (Figure 4b–d) and represents the highest frequency at which the diode can still operate. The parameters that can be extracted from the real part describe the effective resistance (R_ES_) [31] without considering resistance losses due to the junction depletion region. Therefore, the impedance values are significantly lower than the values extracted from the DC measurements (Table S3) and do not strictly follow a trend with increasing nanogap width. Figure S14 shows the RF current distribution simulations, which provide key insight into this phenomenon. As the nanogap width increases, the current profile is confined near the probing area of the GSG (Ground‐Signal‐Ground) probe and does not propagate uniformly, indicating the existence of a possible resonance. The 3 mm diodes exhibited *f*_c‐int_ of 21 GHz, while the 0.95 and 0.63 mm diodes showed *f*_c‐int_ much higher than our setup's measuring limit of 67 GHz. To overcome this issue, we constructed an equivalent circuit to match the diodes’ S parameters and simulated their performance up to 250 GHz. Further details are provided in the Experimental Section and Figure S15. The Smith charts presented in Figure 4b–d and Figure S16 show that the simulated S‐parameters agree with the experimental results. The simulations also show that the smaller diodes with widths of 0.95 and 0.63 mm exhibit *f*_c‐int_ in excess of 131 and 184.6 GHz, respectively. The higher *f*_c‐int_ is attributed to the reduced series resistance (*R_s_*) and junction capacitance (*C*_j_) shown in Figure 4e,f and summarized in Table S3. These results highlight the ability to adjust the diode's operating characteristics, including its frequency response. Notably, the extracted capacitance and low‐frequency resistance values for the 0.63 mm and 0.95 mm diodes are comparable; however, the f_c‐int_ still differs significantly. These variations arise from additional high‐frequency parasitic not captured by the lumped‐element model. Specifically, both parasitic inductance and frequency‐dependent resistance are linked to the device width and the geometry of the inner electrode, becoming increasingly significant at high frequencies and contributing to shifts in the real‐imaginary impedance crossover point that defines f_c‐int_ [46].

**FIGURE 5:**
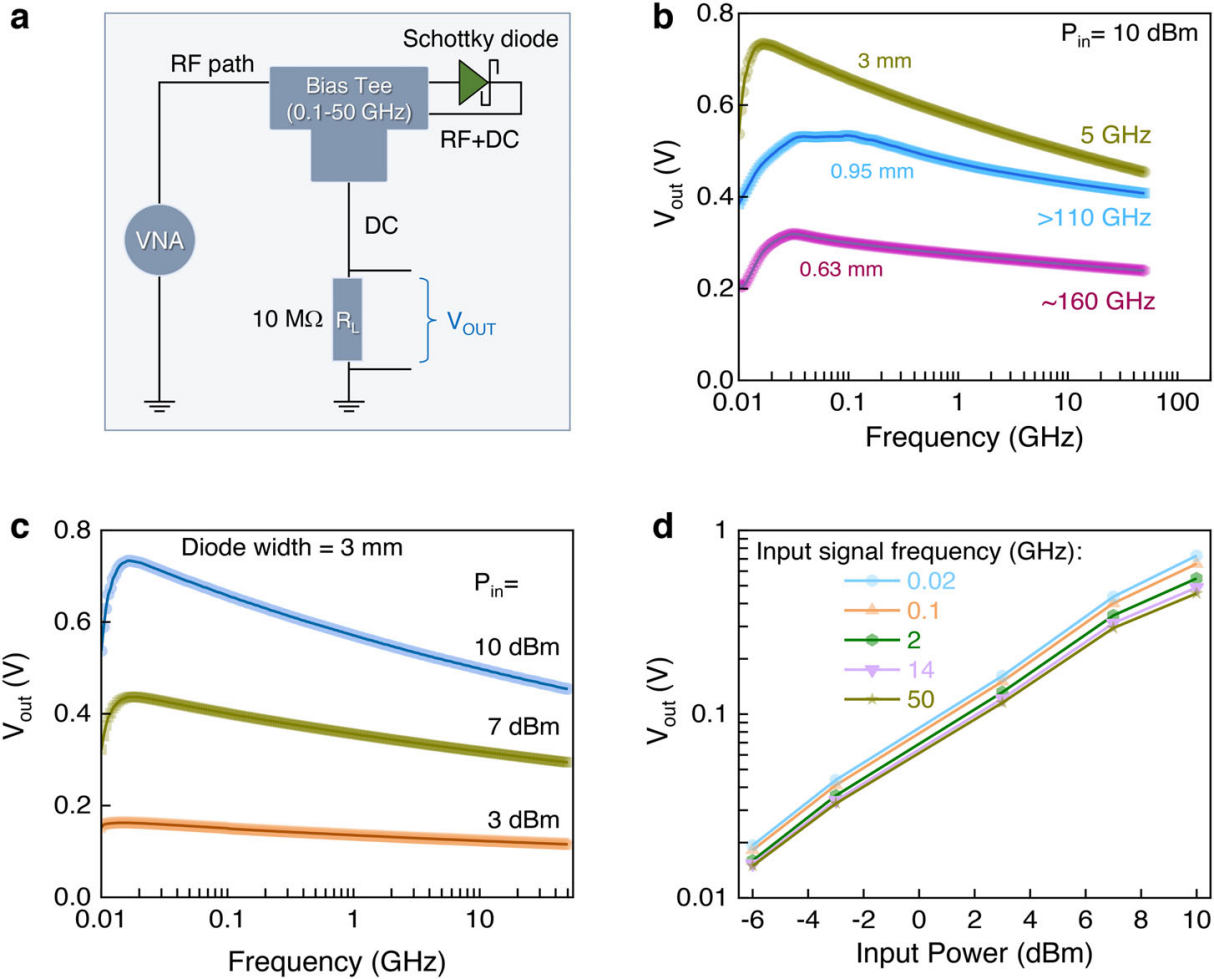
Output voltage at high frequency rectifier measurements. a) Schematic representation of the rectifying circuit, which includes a high frequency signal generator, a bias‐tee and a digital multimeter with a 10 MΩ internal resistance. b) Rectified voltage output dependence on the channel width of the IGZO diodes. The measurements were carried out with P_in_ =10 dBm and the extrinsic cut‐off frequency was estimated at the half power point. c) Rectified voltage output (V_OUT_) of the 3 mm diode as a function of frequency measured at input powers (P_in)_ of 3, 7, and 10 dBm. d) Input frequency dependent voltage output of the 3 mm diodes.

A half‐wave rectifier circuit was realized to evaluate the extrinsic cut‐off frequency (*f*_c‐ext_) of the IGZO diodes. By measuring the direct current (DC) output voltage as a function of input signal frequency, the *f*_c‐ext_ can be estimated from the −3 dB point, i.e. the point at which the DC output voltage (V_OUT_) is equal to V_PEAK_ $/\sqrt{2}$ [47], where V_PEAK_ is the peak DC output voltage. Figure 5a shows the bias‐tee and a 10 MΩ representing the input resistance of the digital multimeter used to measure the V_OUT_. Schottky diodes with larger channel widths yield higher voltage outputs (Figure 5b), with the 3 mm‐width diodes showing the highest V_OUT_ of 0.74 V and a *f*_c,ext_ of 5 GHz. The smaller diodes (0.95 and 0.63 mm) yield *f*_c‐ext_ >110 and >160 GHz, respectively, estimated via linear extrapolation. Although this method does not fully capture the diode behavior across all input signal powers, our measurements remain reliable down to ‐6 dBm. Below this power level, the output signal approaches the noise floor of our setup, making further analysis unreliable. Despite this limitation, the obtained *f*_c‐ext_ values surpass all previously published data for Schottky diodes manufactured via scalable patterning methods [3, 13, 14, 31, 47, 48, 49, 50]. The improved performance is attributed to the ZnO interlayer, which boosts electron injection by lowering the barrier at the Al/ZnO‐IGZO interface by preventing the formation of a native Al_2_O_3_ on the Al surface, which can act as an additional resistive component. Crucially, our IGZO diodes can sustain high input RF power (Figure 5c), with a near‐linear dependence on the input power (*P_in_)* (Figure 5d). Together, these advancements underscore the importance of the scalable contact engineering approach with nanoscale fabrication precision, highlighting its potential to further boost diode performance.

## 3 Conclusion

In conclusion, we developed wafer‐scale planar IGZO Schottky diodes with an unprecedented cut‐off frequency of 160 GHz. Key to this was the development of asymmetric electrode nanogaps (<20 nm in length) featuring contacts with discreetly engineered work functions; one designed to facilitate electron injection while the other to form a large Schottky barrier (0.58 eV) upon contact with IGZO. Due to its fully planar architecture, the ensuing diodes exhibit low junction capacitance (<15 pF) and series resistance, resulting in a maximum estimated intrinsic cut‐off frequency of 184.6 GHz. Half‐wave rectifying circuits made using these IGZO diodes produced promising output voltages up to 0.74 V while being able to sustain high input RF power. The unprecedented operating frequency of these IGZO diodes and their compatibility with scalable manufacturing make our technology attractive for application in wireless energy harvesting and next‐generation 6G telecommunication technologies.

## 4 Experimental Section

### 4.1 Fabrication of Planar Nanogap Structure

Starting, 4‐inch Borofloat glass wafers of 1 mm thickness (from Instruments Glasses Ltd) were used as the substrates of our devices and cleaned sequentially with DI water, acetone, and isopropanol (IPA) under sonication for 10 min in each solvent. Then, the positive photoresist AZ5214 E was patterned via photolithography and 90 nm of aluminum (Al) were thermally evaporated in a high vacuum (2 × 10^−6^ Torr) at the rate of 0.5 Å s^−1^. Next, without exposing the substrate to the ambient environment to avoid alumina (Al_2_O_3_) formation, 10 nm of zinc oxide (ZnO) were sputtered in high vacuum (5 × 10^−6^ Torr) via radio frequency magnetron sputtering at the rate of 0.1 Å s^−1^, resulting in the formation of a bilayer of Al/ZnO which serves as the first metal (M1) of the devices. The patterns were realized using a brightfield mask, removing excess M1 through the lift‐off process using acetone. The patterned substrates, were immersed in an 1mm solution of octadecylphosphonic acid (ODPA, purchased from Sigma–Aldrich) in IPA for 24 h. During that time a selective ODPA SAM is formed on the surface of M1, rendering it hydrophobic but leaving the glass surface without SAM. The substrates were rinsed with IPA and dried with nitrogen gas to remove any physiosorbed ODPA molecules and then annealed at 88^°^C for 15 min to remove any excess solvents. Next, the M2 contact which consists of an Al underlayer (5 nm) to promote adhesion to the glass substrate and an Au layer (93 nm) were thermally evaporated in high vacuum (2 × 10^−6^ Torr) at the rates of 1 and 0.5 Å s^−1^ respectively. The substrates were immersed in acetone for 90 min at a temperature of 90°C and then sonicated for 10 min in acetone and IPA respectively. Because of the inadequate adhesion and internal stress arising from M2 films on M1/SAM surfaces, this process was sufficient to selectively remove the M2 layer. A final step of photolithography and wet etching was carried out to pattern the previously global M1 electrode, thus creating the patterns of fully separated diodes. Last, the wafers were immersed in acetone to remove the excess photoresist and sonicated in acetone and IPA for 10 min respectively. Last, to remove the remaining SAM from the M1 surface and any remaining photoresist an Argon plasma treatment was carried out for 10 min revealing empty nanogap electrodes with a typical gap size of less than 20 nm. To investigate the yield and the electrical reliability of the nanogaps, we measured 100 empty coplanar nanogaps (i.e., without IGZO) (Figure S18a). Among these, only one device exhibited a short circuit (reaching the compliance current of 0.1 A), one showed partial conduction (∼10^−^⁵ A), and two devices displayed minor leakage currents around ∼10^−^⁷ A, resulting in a 96% yield of completely electrically isolated nanogap electrodes. Additionally, a low‐magnification SEM image (Figure S18b) highlights the structural uniformity of the nanogaps across large areas, rendering adhesion lithography a reliable fabrication approach for large area electronics.

### 4.2 IGZO Precursor Preparation and Schottky Diode Fabrication

Initially, solutions of Indium (III) nitrate hydrate (99.999% purity from Sigma–Aldrich), Gallium nitrate (III) hydrate (99.999% purity from Sigma–Aldrich) and Zinc nitrate hexahydrate (purchased from Fisher chemicals) were dissolved separately in 2‐methoxy ethanol at a concentration of 0.1 M and stirred overnight at 1100 rpm. Following, the three solutions were mixed at a volume ratio of (5: 1: 3) and then stirred at 1100 rpm for 6–8 h, forming the IGZO precursor. Then, the substrates were treated with Argon plasma for 8 min to achieve surface activation. After filtering the precursor via a 0.45 µm PTFE syringe filter, the spin coating step was carried out at 2000 rpm for 30 s on top of the nanogap devices. Subsequently, the devices were dried inside a nitrogen filled glovebox at 140°C for 15 min to remove excess solvent. Finally, rapid photonic curing was conducted to convert the precursor using the Novacentrix Pulse Forge 1300. The conditions were optimized based on the delivered energy of the flash which varied from 3 to 6 J/cm^2^. More specifically, the optimal performance of the diodes was achieved after delivering 20 pulses at a voltage of 656 V, with a pulse duration of 630 µs and a firing rate of 1.2 Hz, which resulted in a delivered energy density of 6 J/cm^2^.

### 4.3 Opto‐Thermal Calculations and Temperature Profile Simulations

Opto‐thermal simulations were conducted using COMSOL transient simulator to determine the flash annealing (FLA) parameters and their correlation to the temperature rise on the contacts during the process. The optical response of each electrode was acquired from SOPRA database. A train of 20 pulses with flash‐light power of F ═ 6 J/cm^2^, duration of τ_p_═630 µs, and frequency of *v* ═1.2 Hz was found to be the most favorable for the conversion process.

### 4.4 Electron Microscopy

Top‐view Scanning Electron Microscopy was carried out using the Helios 5 UX microscope, to obtain high‐resolution images of several Al‐ZnO ‐ Al/Au nanogaps. The operating voltage ranged from 2 to 5 KV and the images were acquired using the immersion mode. For the cross‐sectional images, a thin lamella was prepared and extracted using the focused ion beam (FIB) and the nanomanipulator of a Helios 5 UX SEM. We note that the lamella preparation can introduce some artefacts at the nanogap interface. Next, a protective bilayer of carbon and tungsten was deposited, followed by a sample milling with a gallium (Ga) ion‐beam. Then the lamella was cut via the FIB and was soldered on the copper TEM grid with the nanomanipulator. Following, the obtained specimen was thinned down and polished with the FIB, to remove any excess material and avoid potential contamination. The high‐resolution TEM images were acquired using a FEI Titan Themes Cubed G2 300 (Cs Probe) microscope at an operating voltage of 300 kV. Finally, the elemental composition of the sample was carried out by Energy Dispersive X‐ray Spectroscopy (EDX) mapping in the scanning mode of the TEM (STEM).

### 4.5 Energy Levels Assessment

The work function measurements were carried out using a Kelvin Probe Veeco AFM from Bruker in non‐contact mode using Bruker SCM‐PIT‐V2 probes. A fresh Al/Au film (thickness of 5/93nm) on a glass substrate was used as a reference for the calibration of the tip. Subsequently, both the pristine nanogap electrodes and the ones with the fully treated IGZO film were measured separately. Finally, the surface potential difference was used to estimate the work function of the films. The Al/ZnO contacts exhibited a Fermi level of ∼4.24 eV, while the IGZO films showed a slightly deeper surface potential, consistent with a Fermi level near ∼4.34 eV. UPS measurements were carried out using a multi‐probe Scienta Omicron UHV system operating at a base pressure of 5 × 10^−10^ mbar. A (HIS‐13 focus) Vacuum UV source was employed with the intensity attenuated. The UPS electron analyzer is an Argus CU with a rectangular field of view of 4 × 2 mm^2^, (aperture 5, high magnification). UPS spectra were recorded with a 20 and 5 eV constant pass energy respectively. Samples were measured using a 10 eV bias, without charge neutralization, and contacted to the sample via molybdenum strips. All measurements were conducted with the sample plane normal to the direction of the analyzer, with a 0° take‐off angle.

### 4.6 Electrical Characterization

The IGZO Schottky diodes electrical characterization was caried out inside a nitrogen‐filled glovebox using a Keysight B2912A precision source meter. The capacitance measurements were realized using a Keysight B1500A semiconductor device analyzer.

### 4.7 Radiofrequency Measurements

The one‐port scattering parameter (S11) measurements were conducted in the frequency range of 0.01 to 67 GHz under ambient conditions using a Vector Network Analyzer (ME7828A) from Anritsu connected to a Cascade Microtech probe station. Cascade Infinity GSG probes (i110) with a pitch of 150 µm were utilized, along with Gore Phaseflex cables (110 GHz). Last the system was calibrated using a valid Open, Short and Load (OSL) technique on an impedance standard substrate (ISS) of 104‐783. The rectifier circuit measurements were carried out using an Agilent Network analyzer (PNA N5225A) connected to a bias‐tee (0.03 MHz to 70 GHz) purchased from Pasternack which extended to the GSG probe (i110). Last, the output voltage was measured by connecting a Keysight 34465 A digital multimeter with an internal resistance of approximately 10 MΩ to the tee‐bias’ DC output.

### 4.8 RF Current Profile Simulations and Equivalent Circuit Models

The exact geometry of the devices and the properties of each component were taken into account and the currents’ profile is plotted in Figure S14. Equivalent circuit models of the diodes are illustrated in Figure S15 based on the existing literature [51, 52]. It is noted that the additional CPW line part has been included into the circuit model of the 3mm diode compared to the smaller ones (W═ 0.63 and 0.95 mm respectively). Advanced Design System (ADS) has been used for the equivalent circuit simulation. As shown in Figure S16 the simulated S parameters are fairly matched with the measured results in the format of Smith chart. Electromagnetic simulations of the Schottky diodes were carried out using ANSYS High‐Frequency Simulation Software (HFSS).

## Author Contributions

T.D.A. and L.P. outlined the project. T.D.A. and H.F. guided and supervised the project. L.P., L.L., H.F.M.M., and W.S.A. fabricated wafer scale nanogap devices. L.P., H.F., and L.L. performed electrical measurements and analyzed the results. L.P. and H.F. carried out the C‐V measurements. L.P. and H.F. analyzed the C‐V and I‐V data. L.P. and L.L. performed and optimized the flash‐light annealing experiments. S.D. and E.L. performed the opto‐thermal simulations and relevant analysis. L.P., L.L., and T.M. performed electron microscopy measurements. D.N. performed the Kelvin Probe AFM images and analyzed the data. S.M. performed the AFM topography measurements. S.F., and G.T.H. performed and analyzed the UPS measurements. U.S. and M.G. carried out the DFT calculations. A.S. Y.Y., L.P., and H.F., setup the high frequency measurements. L.P. and Y.Y. analyzed the one‐port high frequency measurement results. L.P. and Y.Y. built the equivalent circuit model for the one‐port high frequency measurements. L.P., H.F., and Y.Y. performed and analyzed voltage output measurements. L.P. and Y.Y. performed HFSS simulations. T.D.A and L.P. outlined and drawn the schematics. L.P. wrote the first draft of the manuscript. All the authors discussed the results and contributed to the final version of the manuscript.

## Conflicts of Interest

The authors declare no conflicts of interest.

## Supporting information




**Supporting file**: smll72140‐sup‐0001‐SuppMat.docx

## Data Availability

The data that support the findings of this study are available from the corresponding author upon reasonable request.
