# Supplementary material for: 160 GHz Schottky Diodes from Solution‐Processed IGZO
Source: Small. 2025 Dec 29;22(10):e04148. doi: 10.1002/smll.202504148 (PMC12910416; doi:10.1002/smll.202504148)
Supplement: Supplementary file 1 — Supporting file: smll72140‐sup‐0001‐SuppMat.docx [file SMLL-22-e04148-s001.docx]

# **Supporting Information**

**160 GHz Schottky Diodes from Solution-Processed IGZO**

Lazaros Panagiotidis^1^, Hendrik Faber^1^, Yiyang Yu^2^, Spyridon Doukas^3^, Linqu Luo^1,4^, Mohammed Ghadiyali^1^, George T. Harrison^1^, Dipti Naphade^1^, Suman Mandal^1,4^, Wejdan S. Alghamdi^1^, Harold F. Mazo-Mantilla^1^, Temur Maksudov^1^, George S. Pappas^1^, Udo Schwingenschlögl^1^, Shadi Fatayer^1^, Elefterios Lidorikis^3^, Atif Shamim^2^ and Thomas D. Anthopoulos^4^

^1^ Physical Science and Engineering Division, KAUST Solar Center (KSC), King Abdullah University of Science and Technology (KAUST), Thuwal 23955-6900, Saudi Arabia.

^2^ Computer, Electrical and Mathematical Science and Engineering Division, King Abdullah University of Science and Technology (KAUST), Thuwal 23955-6900, Saudi Arabia.

^3^ Department of Materials Science and Engineering, University of Ioannina, Ioannina 45110, Greece.

^4^ Henry Royce Institute, Photon Science Institute, Department of Electrical and Electronic Engineering, The University of Manchester, Manchester M13 9PL, UK.

**This file includes:**

Supporting Texts

Supporting Figures

Supporting Tables

**ST 1: Self-Peeling of M2 after stress induced from low-temperature annealing**

The peeling-off step is essential for the fabrication of the nanogap electrodes in terms of nanogap quality, size and yield. This step was carried out in the past by either using adhesive tape or an adhesive glue to remove the M2 from the M1-SAM interface. ^[1–5]^ The manual peel-off process though, may lead to non-uniform nanogap electrodes which may render this step imprecise. Furthermore, the application of adhesive glue or tape introduces the risk of contaminating the nanogap electrodes with residual substances. Loganathan et al. ^[6]^ proposed a self-forming nanogap electrode by using a Ti/Pt bilayer as M2, due to the fact of the spontaneous delamination of M2 from the area where the SAM is present. In this study, we managed to introduce a new peel-off step which can be applied to any material deposited as M2. This step includes temperature treatment of the substrates to modify the SAM and organize it on the M1 surface at will. In particular, post-thermal treatment plays a crucial role in enhancing the bonding between ODPA and the metal oxide as was formerly discussed. Interestingly, our observations indicate that when the substrate is placed in vacuum for 120 minutes before the deposition of the M2, the subsequent peel-off process becomes significantly easier. Additionally, after the deposition of M2 we immerse the wafers in DI water, acetone, Isopropyl Alcohol (IPA) or N-Methyl-2-pyrrolidone (NMP) for 90 minutes at 90 °C aiding the self-peeling-off of M2 by increasing the internal stresses of the film. Through temperature the mechanical stresses are delivered to the overlapping areas between the M2 and M1/SAM surface, resulting in instantaneous delamination. The substrates were then sonicated for 5 minutes in Acetone and IPA respectively, to remove any peeled-off excess and reveal the coplanar nanogap electrodes. The self-formed nanogaps show uniformity and repeatable channel lengths (<20 nm).

**ST 2: Geometric consideration**

The Al/ZnO-Al/Au diodes designs are shown in Figure S1. The specific geometrical parameters are also shown and fully defined. The channel length (L) refers to the distance between the two contacts. The perimeter of the inner electrode represents the channel width (W) which in this work designed to be 3 mm, 0.95 mm and 0.63 mm. The electrodes’ thickness denoted as height (H) was always kept at 100 nm. The active area of the nanogap devices is determined by multiplying the width (W) by the thickness (H) and is found to be 63 μm^2^, 95 μm^2^ and 300 μm^2^ for each corresponding width. To efficiently probe the devices with GSG picoprobes we incorporated a notch into our design for the 3 mm diodes, as shown in Figure S1a. Smaller diodes with W= 0.95 mm and 0.63 mm could be probed without the need of the notch, hence we kept the simplified circular geometrical approach.

**ST 3: Nanogap length (L) extraction**

To extract the average nanogap length of the coplanar nanogap devices we have employed two different methods recommended in the existing literature.^[5], 7]^

**Method 1:**

We utilized the ImageJ software to load high-resolution scanning electron microscopy (SEM) and atomic force microscopy (AFM) images, both illustrating the coplanar a-lith nanogaps. The embedded scale bar of each image was used to establish the conversion factor from pixels to nanometers. Within the software, we manually measured the gap size multiple times across the nanogap, as demonstrated in Figure S2 a and c. Each white vertical line in the image represents one measurement, with the vertical length of each line indicating the nanogap length. This method involved a manual placement of approximately 200 equidistant vertical bars to determine both the mean gap size and its distribution.

**Method 2:**

The second method, was based on a digital image analysis technique proposed by Kano et al.^[7]^, to ascertain the average distance of the nanogap between the two electrodes. Initially, the same high-resolution SEM and AFM images were imported into the ImageJ software and converted into binary images, with black pixels denoting the nanogap space and white pixels representing the surrounding metal electrodes as shown in Figure S2b and d. The extraction of the perimeter (P) involved manually outlining the binary nanogap image within the Procreate image software using a stylus pen. Following the export of the binary image, a Python script was employed to analyze the pixels in each column of the image, specifically examining the number of black pixels present at each x-coordinate and successfully quantify the area (S) and perimeter (P) of the nanogap space in terms of pixels/nm. Those values were employed to calculate the average gap size (D_ave_) based on the Equation S1:

D_ave_ = $\frac{S}{0.5 P}$ (S1)

The gap area S was determined by counting the total number of black pixels within the gap, and the perimeter (P) was calculated by selecting the outer pixels surrounding the nanogap space while excluding those inside it. Consequently, the average gap size was calculated in terms of pixels/nm and then converted into nm using the pixel-to-nanometer conversion factor, as determined in the calibration process outlined in Method 1. It's worth noting that unlike the manual Method 1, the Kano et al. method provided the average gap size but didn't generate a size distribution histogram.

The mean nanogap size determined using both methods, was approximately 20 nm. Both estimations 1 and 2, rely on SEM and AFM images and share similar limitations arising from the constraints of SEM regarding image resolution and local contrast. Achieving precise measurements of features at the nm scale pushes the limits of both imaging methods capabilities. Additionally, the local contrast at the gap edges may not allow for an exact pixel-level determination of the gap outline, and to some extent, this determination depends on the user's judgment. Employing an automated algorithm, as recommended by Kano et al., can assist in this process, but users still need to adjust threshold levels for accurate binary image conversion, resulting in a degree of uncertainty when determining the precise nanogap length. Image J was utilized to manually extract the nanogap size from the cross-section TEM image shown in Figure S2e showing an average nanogap length of 19 nm.

**ST 4: Density functional theory (DFT) calculations**

All calculations adopted the Vienna ab-initio simulation package ^[8]^, the generalized gradient approximation of Perdew, Burke and Ernzerhof ^[9]^, the Grimme van der Waals correction ^[10]^, the Bengtsson dipole correction ^[11]^, a plane-wave cutoff energy of 450 eV, and a Monkhorst-Pack k-grid with 0.013 1/Å resolution. The structural relaxation was considered to be converged when the Hellmann-Feynman forces had fallen below 0.01 eV/Å for all the atoms. An ODPA molecule was relaxed using Gaussian09 ^[12]^, the B3LYP functional, and the 6-311G(d,p) basis set. The phonon frequencies were used to verify the structural stability. The interaction of the phosphine group of the ODPA molecule (see Figure 1(a) for the electrostatic potential map obtained by Gaussview ^[13]^) with the experimentally stable Al_2_O_3_ (111) and ZnO $\left( 10\bar{1}0 \right)$ surfaces (2 × 2 × 1 supercells) was studied. A vacuum layer of >25 Å thickness was included to build slab models. The binding energy was calculated as difference between the total energies of the combined system and its components. The energetically favorable position of the phosphine group was found to be on top the Al/Zn-O-Al/Zn hollow site with a monodentate bond formed in agreement with Ref. [14]. 10 random IGZO structures were built using Packmol ^[15]^, with an In:Ga:Zn:O ratio of 5:1:3:12. The lowest energy structure after structural relaxation was selected.

**Results**

We obtain binding energies of -2.53 eV for an ODPA molecule on the Al_2_O_3_ (111) surface and -6.45 eV for an ODPA molecule on the ZnO $\left( 10\bar{1}0 \right)$ surface, indicating stronger binding in the latter case. We find a Bader charge transfer of 0.18 electrons from the Al_2_O_3_ (111) surface to the ODPA molecule and a Bader charge transfer of 0.24 electrons from the ODPA molecule to the ZnO $\left( 10\bar{1}0 \right)$ surface, see the charge density difference plots shown in Figure S3b, c. This indicates that the ZnO $\left( 10\bar{1}0 \right)$ surface is better suitable for binding ODPA, thereby enhancing the quality of the formed nanogap in the experiment. Furthermore, partial densities of states calculated for the IGZO-Al_2_O_3_ (111) and IGZO-ZnO $\left( 10\bar{1}0 \right)$ interfaces, as shown in Figure S4a, b, demonstrating a band gap in the former case and a metallic character in the latter case.

**ST 5: Opto-thermal simulation**

The optical response of each electrode, under normal incidence, was acquired using the transfer matrix method ^[16]^. The optical constants of Au, Al, ZnO_x_ and glass materials were taken from the Sopra database ^[17]^. For simplicity, the optical properties of the dried IGZO precursor were assumed as for the glass substrate. The material thicknesses for each type of contact were assumed as follows: a) Glass substrate (1.1 mm), Al (5 nm), Au (93 nm) and b) Glass substrate (1.1 mm), Al (90 nm), ZnO_x_ (10 nm). In both cases, the IGZO precursor thickness is estimated between 5 – 10 nm, so a median value of 7.5 nm was assumed in the TMM calculations. The absorptivity of each contact is plotted in Figure S8a.

During the FLA process, 20 short pulses of duration τ_p_ = 630 μs with a frequency of $v$ = 1.2 Hz were utilized. The pulse fluence was chosen at F = 6 J/cm2 as the most favourable for the conversion process. The lamp spectrum is plotted in Figure S8b and the resulting absorbed power spectrum is shown in Figure S8c.

2D axisymmetric heat transfer simulations were conducted using the COMSOL transient simulator. The thermal properties of Au, Al and ZnO were acquired from the Matweb database ^[18]^. The thermal simulations were performed assuming convective (free convection, h_top_=10 W/m^2^K) and radiative cooling from the top surface and convective cooling (loose contact with metal holder, h_back_=150 W/m^2^K) from the back surface ^[6]^. Finally, boundary heat fluxes were assumed at the top of each contact type, assuming a temporal heating profile as for Figure S8d.

Figure 1c of the main text plots the transient temperature rise of each electrode, within the nanogap and at the substrate during FLA. It is apparent that despite the different temperatures developed in the two different types of contacts, arising from the different contact absorptivity of the two electrodes, the temperature rise in either side of the nanogap (noted as β_Au_ and β_Al_ for the Au and AlZnO side respectively) is almost identical, for gap length below 100 nm. This result highlights the suitability of the FLA process for the IGZO conversion and its homogeneity inside the nanogap. At the same time, the substrate, given its larger volume and the short pulse duration, stays at low temperatures, with a peak temperature rise ~ 20 K. This fact contributes to the non-destructive nature of the FLA, with most of the deposited power absorbed by the contacts and transferred onto the IGZO precursor.

Overall, the simulations showcase the uniform temperature rise distribution within the nanogap, with peak temperature rise ~ 300 K above ambient temperature. Both the value and the uniformity of this temperature rise demonstrate the suitability of the FLA process for the conversion of the IGZO precursor within the nanogap.

**ST 6: Rectification ratio and responsivity of the diodes**

The rectification ratio of a diode refers to the ratio between the forward and reverse current at equivalent (absolute) voltages, indicating the diode's asymmetry ^[19], [20]^.

Rectification Ratio (*V*) = $|\frac{I_{F}(V)}{I_{R}(V)}|$ (S2)

The (quasi-DC) responsivity, also known as current sensitivity, quantifies the variation in the DC output current corresponding to a specific RF input power. It is calculated in a small-signal approximation by assessing the second derivative of the I-V curve in relation to the differential conductance as shown in the Equation S3.

Responsivity (*V*) =$\frac{1}{2}\frac{\frac{ⅆ^{2}I}{ⅆV^{2}}\left( V \right)}{\frac{ⅆI}{ⅆV}\left( V \right)}$ (S3)

**ST 7: Schottky barrier height, free carrier density, built-in potential, series resistance and ideality factor calculations**

From the C-V measurements we extracted important figures of merit that describe our Schottky diodes, such as the barrier height (*Φ_Β_*), the built-in potential (*V_bi_*) and the carrier concentration *N_A/D_*. The plot of the reciprocal square of the capacitance against the voltage (Mott-Schottky plot, f (V) = $\frac{1}{C^{2}}$) was applied and the capacitance of the empty nanogap electrodes was subtracted, to obtain the corrected values as shown in Figure S9d.

To extract the doping concentration N_A/D_ and built-in potential *V_bi_* which refers to the voltage where no band-bending or charge depletion that separates depletion from accumulation region are present. From the slope of the Mott-Schottky plot (Figure S9f) we apply Equation S4.

$\frac{1}{C^{2}}=\frac{2(V_{\mathrm{bi}}-V-\frac{\mathrm{kT}}{q})}{A^{2}q\varepsilon\varepsilon_{0}N_{A/D}}$ (S4)

where *ε* is the dielectric constant of IGZO and *ε_0_* is the dielectric permittivity in vacuum (ε_0_ = 8.856 x 10^-12^ F m^-1^).

To calculate the barrier height *Φ_Β_* we apply the calculated values of *V_bi_* and *N_A/D_* on Equation S5

Φ_𝐵_ = 𝑉_𝑏𝑖_ + $\frac{\mathrm{kT}}{q}$ (𝑙𝑛 $\frac{N_{CB}}{N_{A/D}}$+ 1) (S5)

where *N_CB_* refers to the effective density of states in the conduction band of the semiconductor and is calculated from Equation S6.

𝑁_𝐶𝐵_ = 2 $(\frac{\text{2πm*kT}}{h^{2}}$ $)^{\frac{3}{2}}$ (S6)

For IGZO, the given values are the following: m* = 0.27m_0_ ^[6],^ ^[21]^ and the calculated is N_CB_ = 3.5×10^18^ cm^-3^.

The diode’s series resistance (*R_S_*), was calculated from the I-V plot, utilizing a method introduced by Cheung et al.^[22]^. As shown in Figure S10a the thermionic emission region is identified, as the intermediate region enclosed by the intersection of the two linear regions. By applying the thermionic Equation S7,

I = I_0_ e^(qV/nKT)^ (S7)

where *I_0_* stands for the reverse bias saturation current and is equal to *I_0_* = 𝑆*𝐴* 𝑇^2^ * $e^{(\frac{q\Phi_{B}}{KT})}$. Here, *S* represents the diode’s area (cm^2^), *n* represents the ideality factor, *Φ_Β_* the barrier height and *A** the Richardson constant equal to A* = 41 A/cm^2^ K^2^ for the IGZO semiconductor ^[6]^. By applying Equation S8 we include the series resistance *R_S_.*

$\frac{\mathrm{dV}}{d(lnI)}$ = $\frac{n}{\beta}$ + $IR_{S}$ (S8)

By calculating β = $\frac{q}{\mathrm{KT}}$ and plotting d(V)/d(lnI) we can extract the series resistance *R_S_* from the slope of the linear fit and the ideality factor *n* from the intersect (Figure S10b). From the Equations S9 and S10 we can obtain a second approximation of the *R_S_* and extract the barrier height *Φ_Β_* from the slope and intersect of the linear fitted H(I)/I plot as shown in Figure S10c.

H(I) ≡ 𝑉 − $\frac{n}{\beta}$ $\ln$($\frac{\text{I}}{A_{\mathrm{eff}}\text{*A*}T^{2}}$) (S9)

H(I) = RI + nΦ_B_ (S10)

**ST 8: Intrinsic and extrinsic cut-off frequency estimation**

Cutoff frequency of a Schottky diode refers to the maximum frequency at which the diode can effectively operate. The intrinsic cut-off frequency (*f_c, int_*) can be measured via one-port S_11_ parameter measurements and describes the operational frequency of the diode without taking into consideration the losses associated with the circuit and the diode itself. The *f_c, int_* can be roughly estimated from the -3dB point from the S_11_ over frequency plot shown in Figure S13. To extract the exact value, we imported the raw data to Matlab and plotted the impedance over frequency plot. The *f_c, int_* is estimated from the intersection point of the real (resistance) and imaginary (reactance) of the impedance. It is important to mention that the *f_c, int_* can provide only an initial understanding of the diodes’ radiofrequency properties providing a theoretical upper limit of the operational frequency.

To include losses associated with impedance mismatching, dielectric losses and reflection we built a rectifying circuit where our diodes are incorporated and proceeded to output voltage measurements to extract the external cutoff frequency (*f_c, ext_*). The same principle for the *f_c, ext_* rough estimation of the -3dB point can also be utilized in that case. Specifically, the -3dB point describes the frequency at which the output voltage *V_OUT_* is reduced by $\sqrt{V_{0}}$ where $V_{0}$ represents the input voltage (low frequency voltage).

## **Supporting Figures**


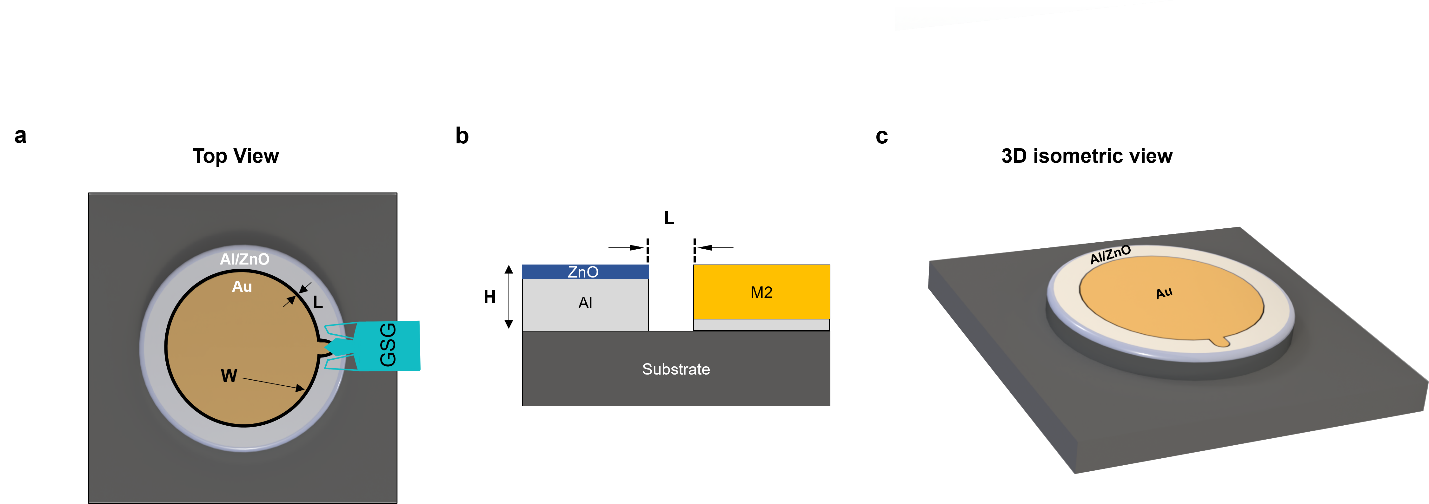


**Figure S1. Schematic (2D and 3D) representation of co-planar Al/ZnO-Al/Au nanogap electrodes.** a) 3D top view of the coplanar showing the corresponding channel width (W) and length (L) of the coplanar contacts. The channel length is estimated from top view SEM, AFM and cross-sectional TEM images. The nanogap width represents the perimeter of the M2 electrode. We used three different channel widths, W = 3 mm, 0.95 mm and 0.63 mm to investigate the impact of the geometry on the devices’ performance in both DC and AC measurements. For the RF measurements we used GSG probes of various pitches (from 150 μm to 250 μm), using M1 as the ground and M2 as the signal. b) 2D cross-sectional view of the nanogap contacts depicting the bilayer structures of M1 and M2, the channel length (<20 nm) and the thickness H (100 nm). c) 3D isometric view of the nanogap contacts.


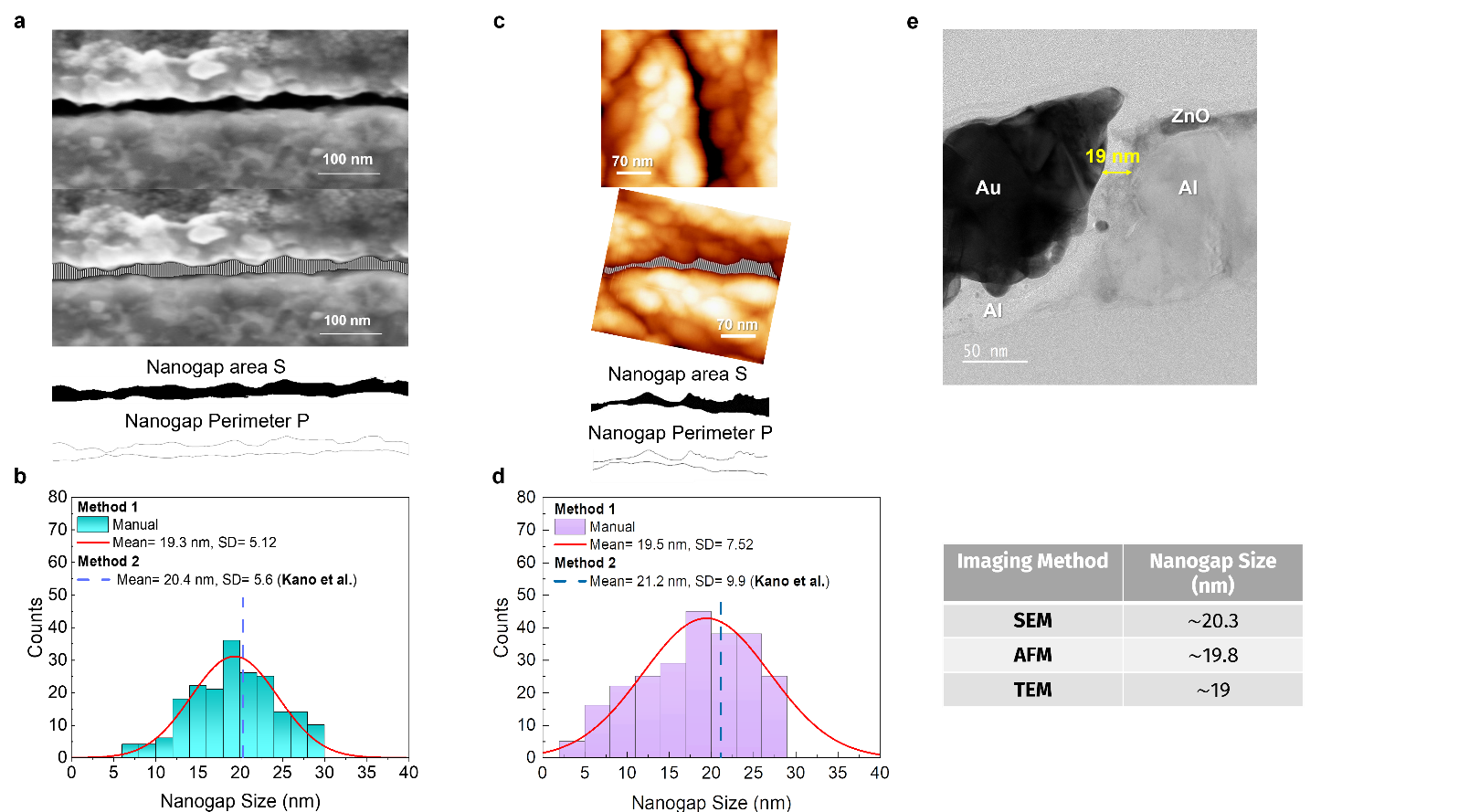


**Figure S2. Average nanogap size extraction**. a) High-resolution top view SEM image displaying the Al/ZnO-Al/Au nanogap used in figure b) to estimate the nanogap size. Two different methods were employed for that estimation. Method 1 refers to the manual estimation of average nanogap size using ImageJ software by considering the equidistance white lines across the nanogap area, while Method 2 refers to the illustrated extraction of nanogaps’ area (S) and perimeter (P) through an analysis adapted from Kano et al. ^[7]^ to calculate the average nanogap size (D_ave_). b) a comparison of the mean nanogap size obtained by these two distinct methods, along with the size distribution of the gaps determined using Method 1 resulting in ~20.3 nm nanogap size. c) An AFM image displaying the Al/ZnO-Al/Au nanogap that is used following the two exact same methods mentioned above. d) comparison of the mean nanogap size resulting in ~19.8 nm nanogap size. e) Cross-section TEM image of the coplanar nanogaps revealing a ~19 nm nanogap size.


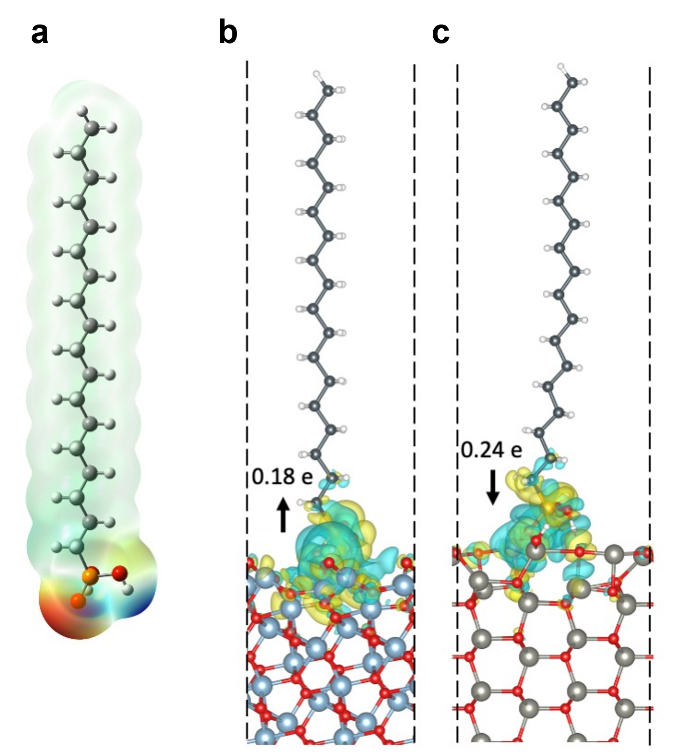


**Figure S3.** a) Electrostatic potential map of an ODPA molecule. Charge density difference plots for an ODPA molecule on the b) Al_2_O_3_ (111) and **c)** ZnO $\left( 10\bar{1}0 \right)$ surfaces. Cyan color represents charge depletion and yellow color represents charge accumulation. The arrows indicate the direction of the charge transfer.


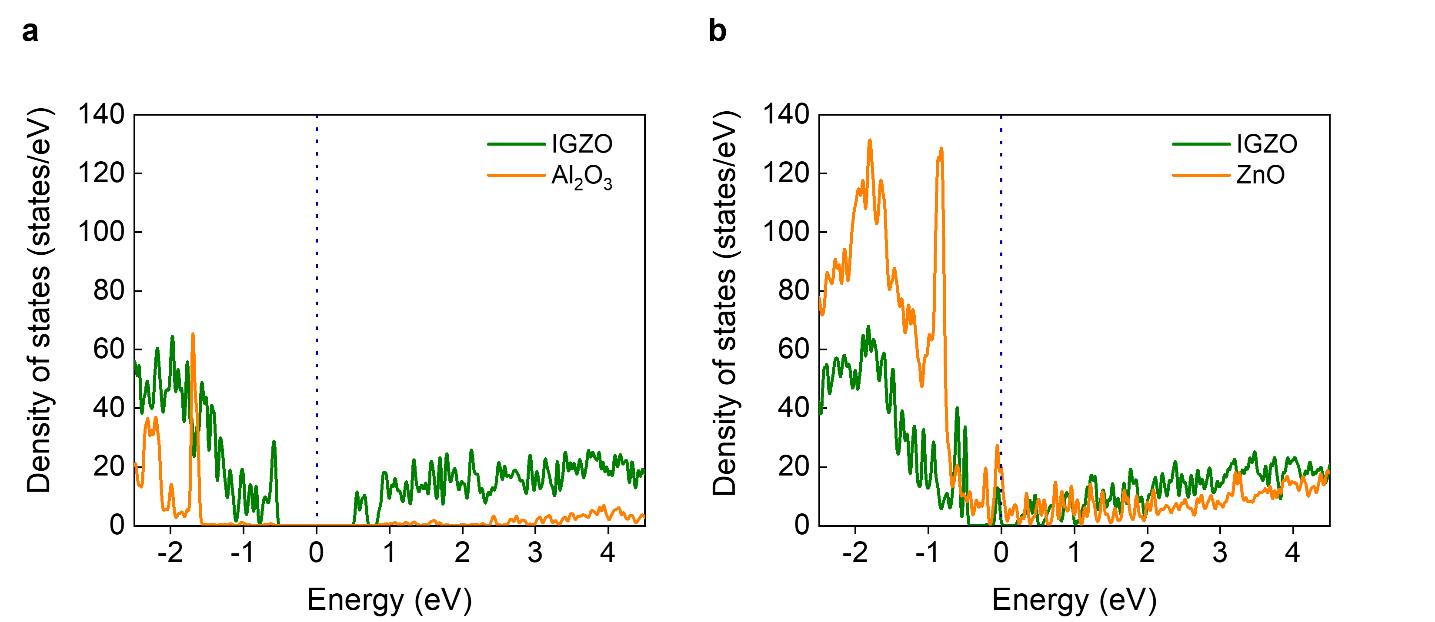


**Figure S4.** Partial densities of states of the a) IGZO-Al_2_O_3_ (111) and b) IGZO-ZnO $\left( 10\bar{1}0 \right)$ interfaces.


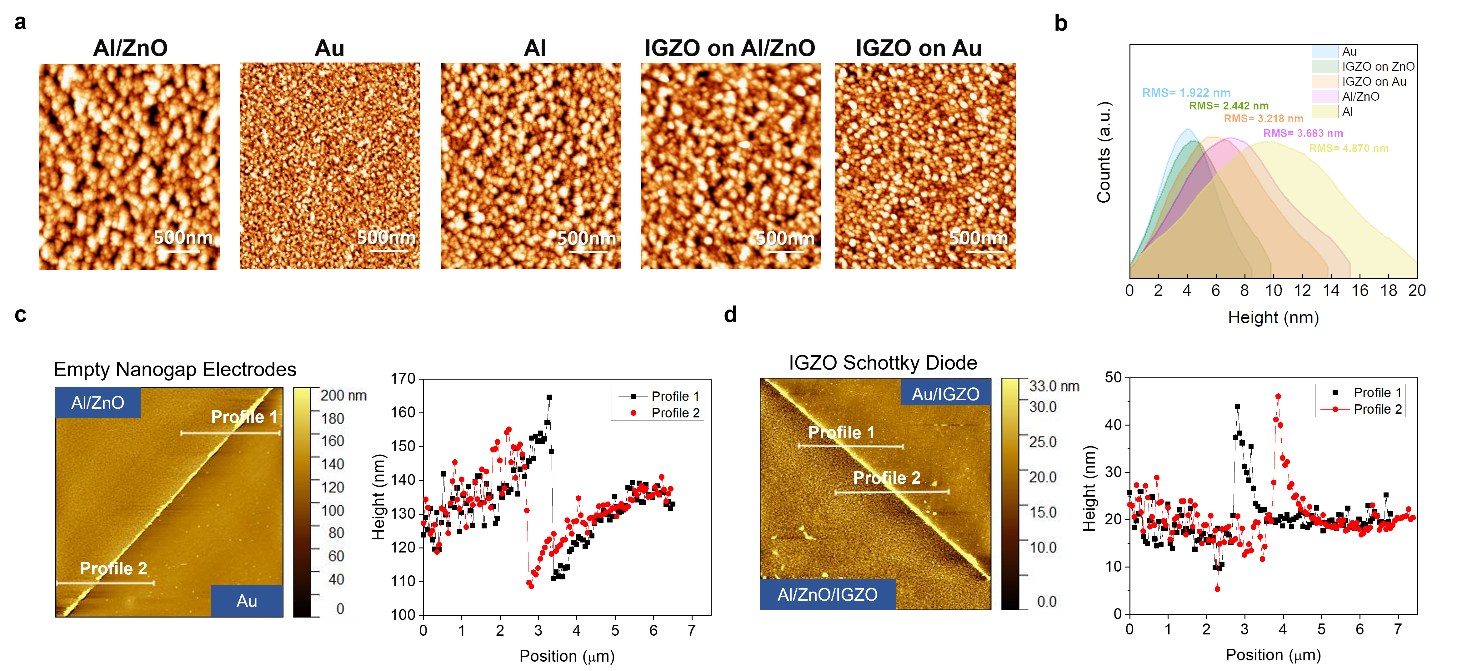


**Figure S5.** a) AFM images of the surface topographies of Al/ZnO, Au, Al and flash-lamp annealed IGZO on Al/ZnO and Au. b) Height distributions extracted from the AFM images of the corresponding measured materials. Topography AFM across the coplanar nanogap with a scan size of 15 x 15 μm and profile plots c) prior the IGZO deposition and d) post the IGZO deposition and flash-lamp annealing step.


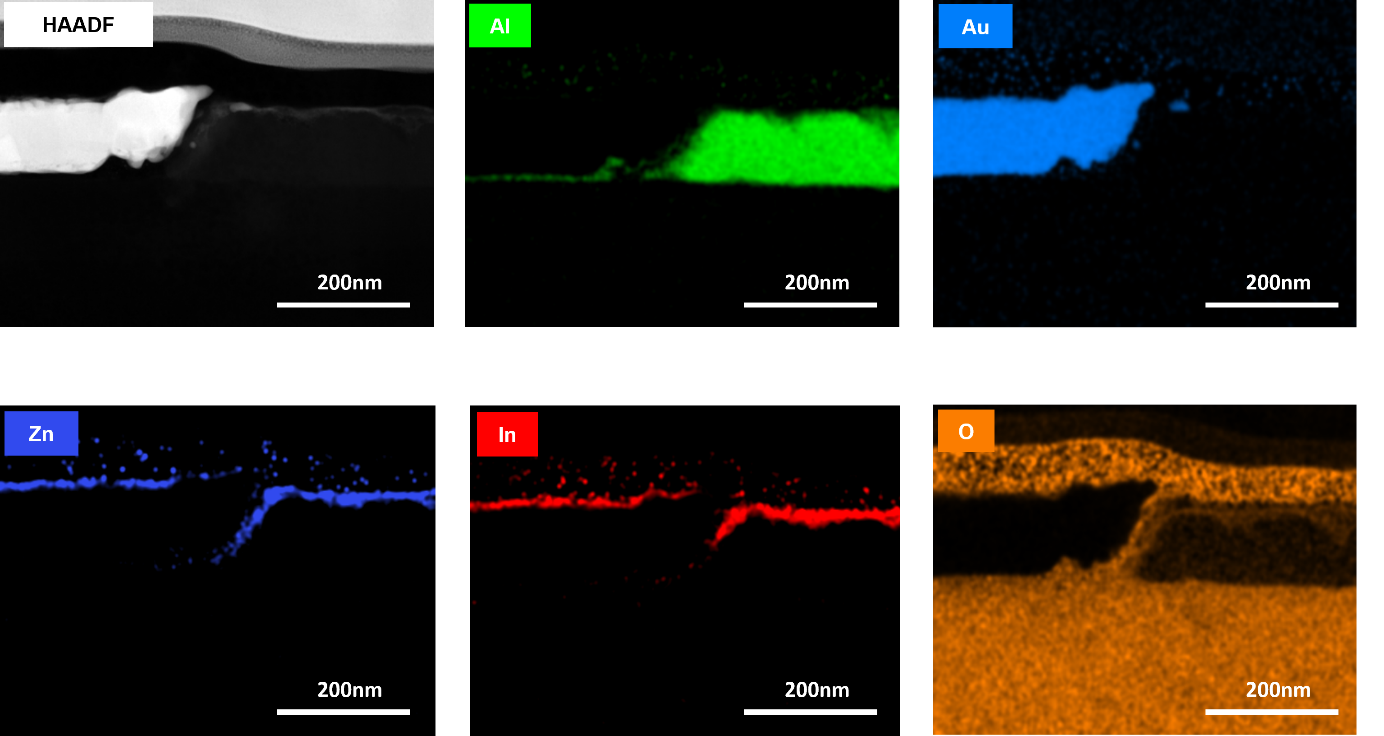


**Figure S6.** High angle annular dark field (HAADF) image and the corresponding elemental mapping of individual Al, Au, Zn, In and O elements.


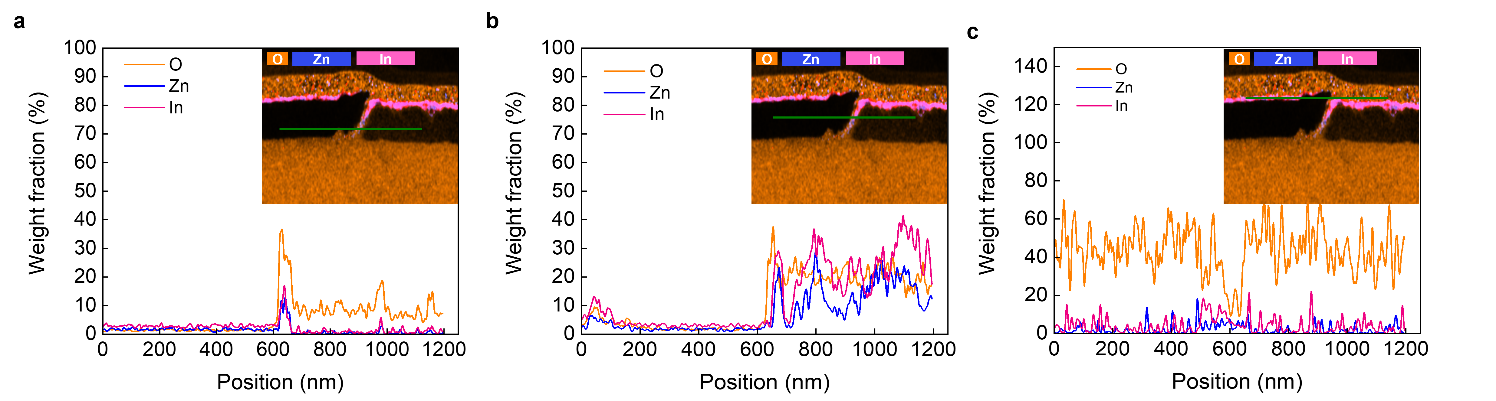


**Figure S7.** a-c) Line profiles of Zn, In and O elements are depicted across the nanogap at different channel heights. The inset images illustrate the combined elemental map of In, Zn, and O with the green lines indicating the regions where the line profiles were taken.


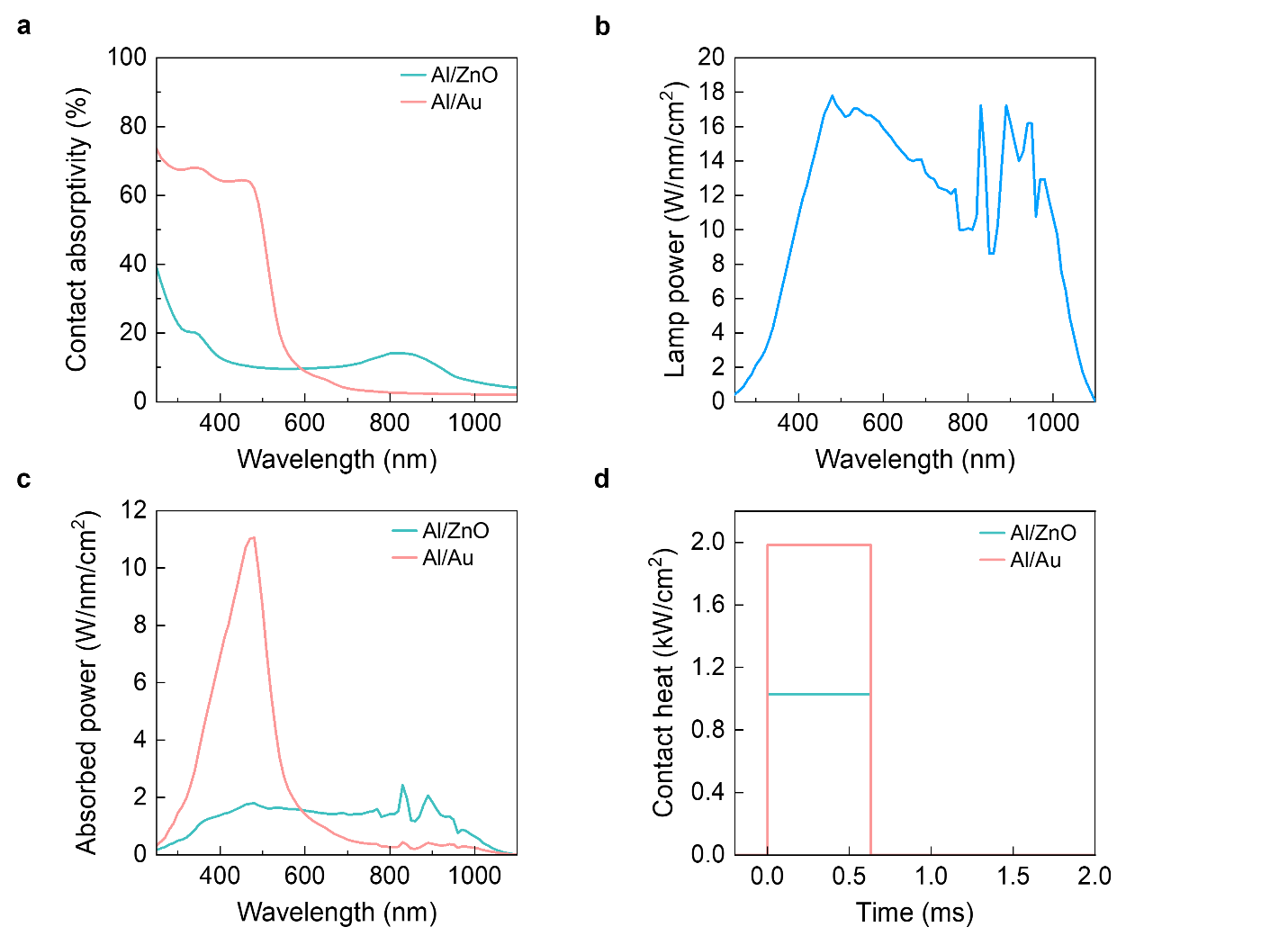


**Figure S8.** a) Contact absorptivity of Al/ZnO and Al/Au contacts. **b)** Xenon flash-lamp power spectrum. **c)** Absorbed power spectrum and **d)** temporal heating profile of each individual contact.


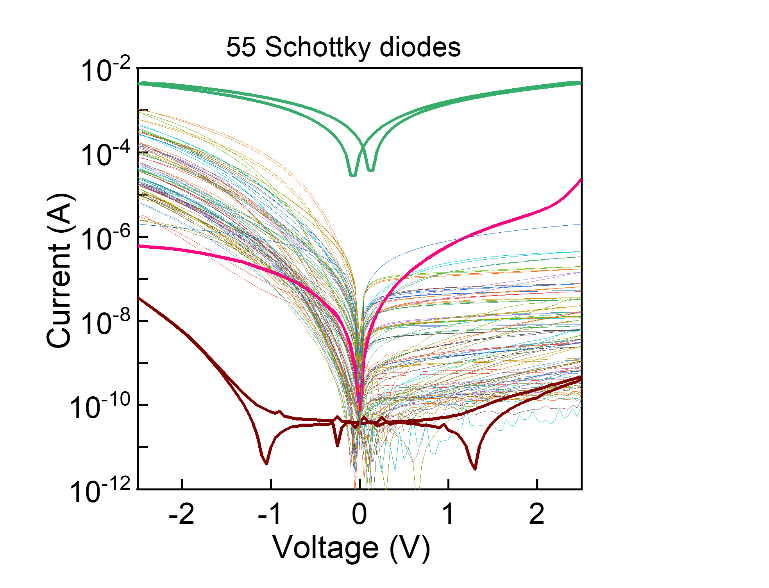


**Figure S9.** Electrical characterization of 55 representative IGZO Schottky diodes from four different wafers, measured after Flash Lamp Annealing (FLA). Devices were selected from various wafer locations and different channel widths (0.63 mm, 0.95 mm, and 3 mm). Among the 55 devices tested, 52 exhibited clear Schottky diode behavior.

**Figure S10. Capacitance – Voltage (C-V) measurements**: a-c) Different width Schottky diode’s C-V curve measured at three different frequencies ranging from 10 kHz to 1 MHz. d) The Mott-Schottky plot for the IGZO diodes where the corresponding empty nanogap capacitance (open circles) the experimental values (filled circles) are shown. e) The corrected Mott-Schottky plot at the three different frequencies after subtracting the empty nanogap capacitance from the experimental capacitance. f) Fitting and extraction of built-in voltage (V_bi_), the doping concentration N_A/D_ and barrier height (Φ_B_).


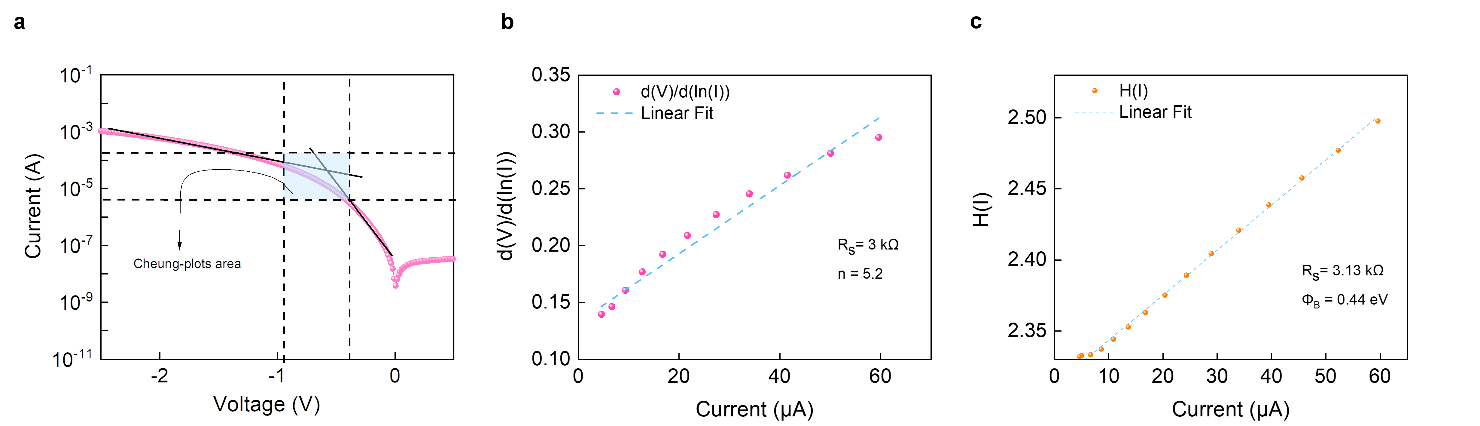


**Figure S11. Extraction of series resistance (R_S_), ideality factor (n) and barrier height (Φ_Β_) from the I-V plots:** a) The thermionic region (I-V range) used for Cheung plots, b) dV/d(ln(I)) vs. the diode’s current for the extraction of ideality factor (n) and series resistance (R_S_) using first-order approximation. c) H(I) vs. the diode’s current plot for the second approximation of R_S_ and the barrier height (Φ_B_) extraction.


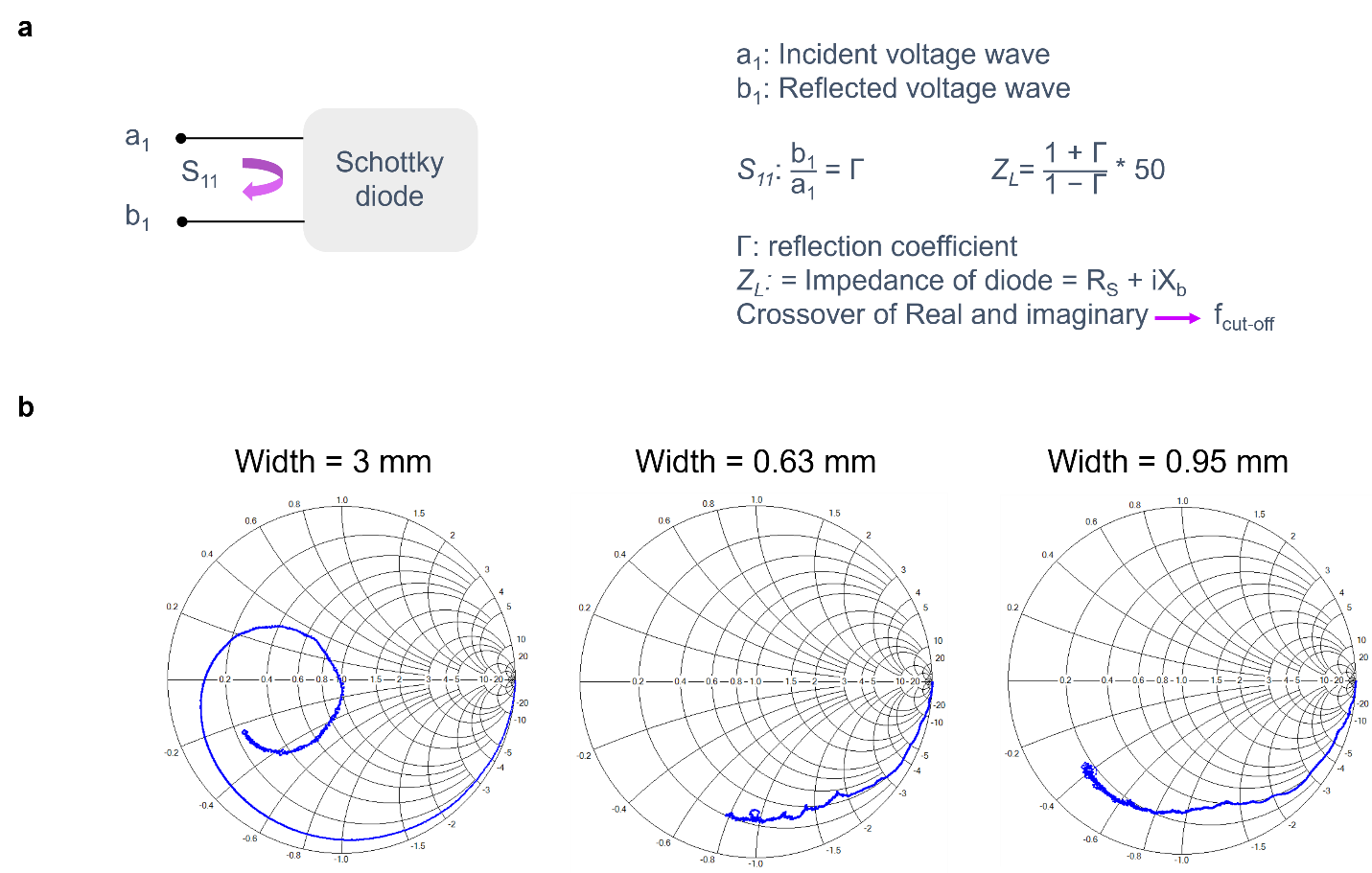


**Figure S12. One-port S_11_ measurements:** a) the schematic illustration of high-frequency one port S_11_ reflection measurements ^[23]^. b) The Smith chart of three different-width IGZO Schottky diodes measured at a frequency range from 10 MHz to 67 GHz showing consistent results. In the lower half of the circle where the plot is displayed, reveals that the reactive component of the Device Under Test (DUT) primarily originates from the junction capacitance (C_j_) of the diode.


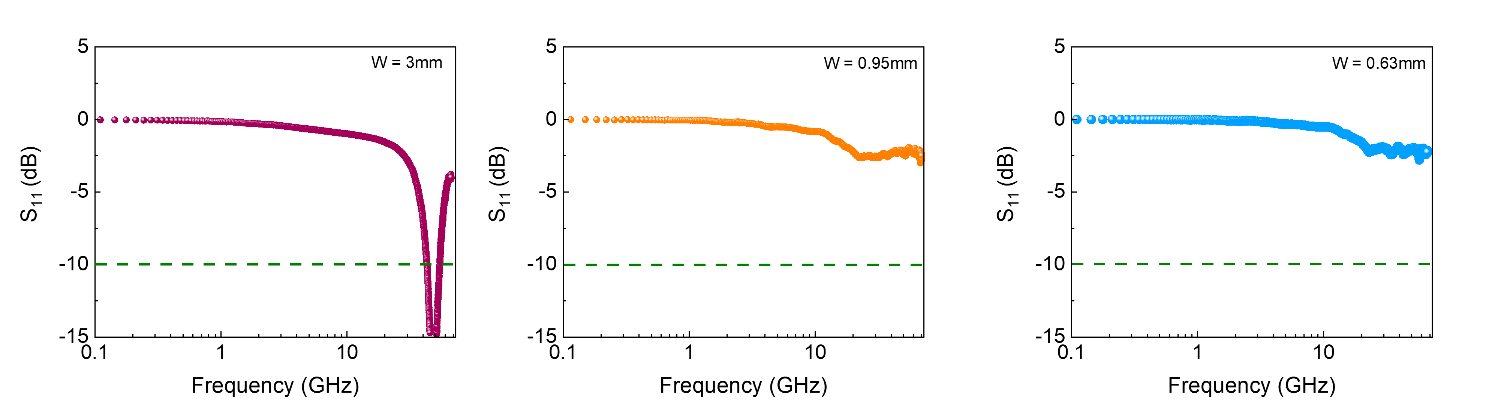


**Figure S13. S11 measurements of the a) W = 3 mm , b) W = 0.95 mm and c) W = 0.63 mm IGZO diodes.** The measurements were carried out at a frequency range from 10 MHz to 67 GHz showing consistent reflection results. The -10 dB point, marked by green dotted lines, signifies where 90% of the input signal is assumed to pass through the device. Corresponding impedance and intrinsic cut-off frequency estimations were carried out and shown in Table S3.


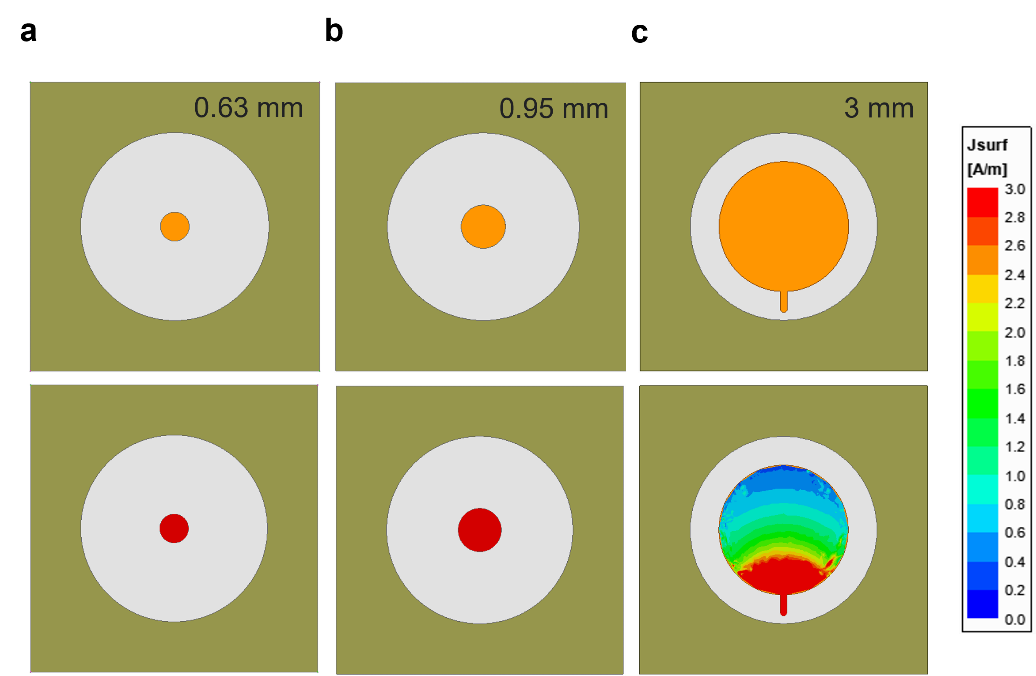


**Figure S14 | ANSYS High-Frequency Simulation Software (HFSS) based of RF current distribution simulation results:** a-c) The bottom row depicts the current map on Al/ZnO-IGZO-Al/Au diodes while launching the RF signals on the 3 mm, 0.95 mm and 0.63 mm diodes. The current distribution for the diodes depicted on a) and b) appears to be uniform. However, in the case of the 3 mm devices c) the current appears to be more concentrated near the probing region and drops with the distance from the probing region. This simulation highlights that as the device structure increases in size, the launch and transmittance of the RF signal becomes challenging.


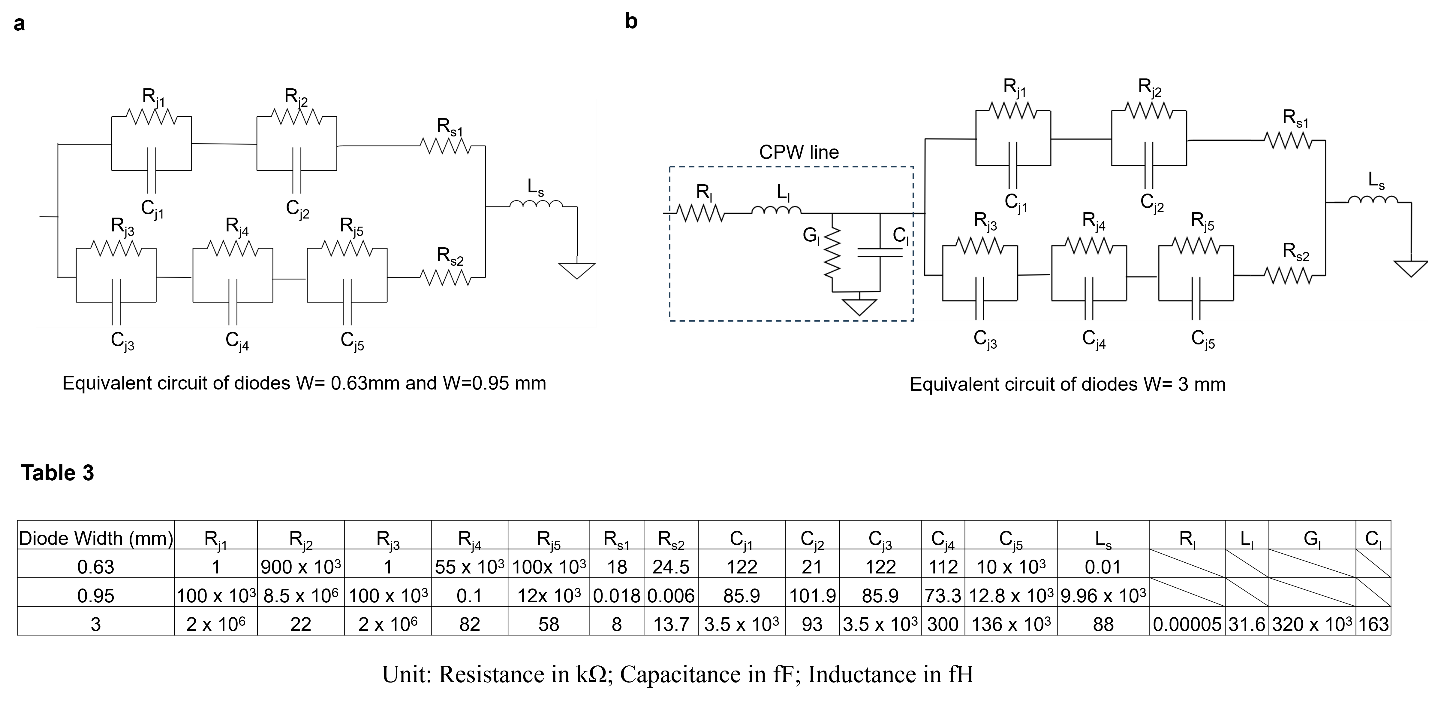


**Figure S15. Equivalent circuit models of the diodes with Advanced Design System (ADS).** a) Equivalent circuit for the 0.95 mm and 0.63 mm. b) Equivalent circuit for the 3 mm diode, with an additional CPW line part which was included into the circuit model to efficiently simulate the experimental S parameters. The table shows all the components’ parameters, used for the simulations.


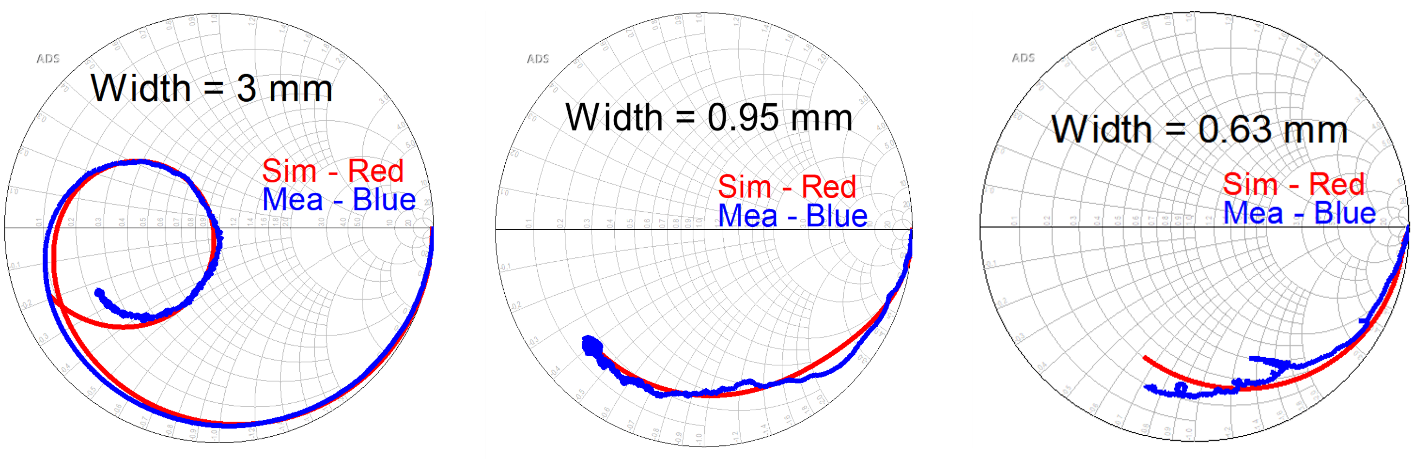


**Figure S16 | Advanced Design System (ADS) simulations of the S_11_ experimental data.** Fairly matched S parameters with the measured data in the format of Smith chart.


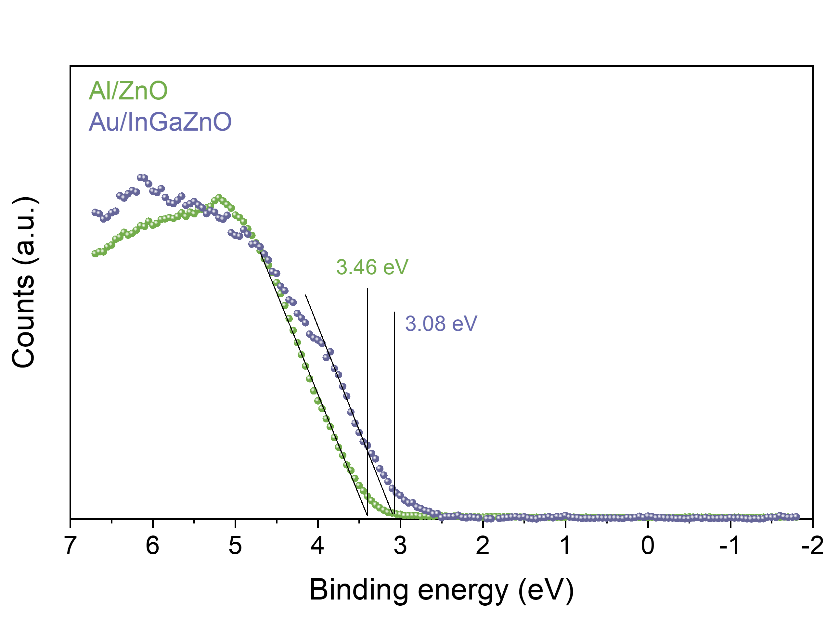


**Figure S17. UPS with a 21.22 eV He (1) line.** Valence band region of the spectrum of ZnO/Al (green trace) and InGaZnO/Au (blue). A linear function is used to estimate the valence band maximum, which corresponds 3.46 eV and 3.08 eV for ZnO/Al and InGaZnO/Au respectively.


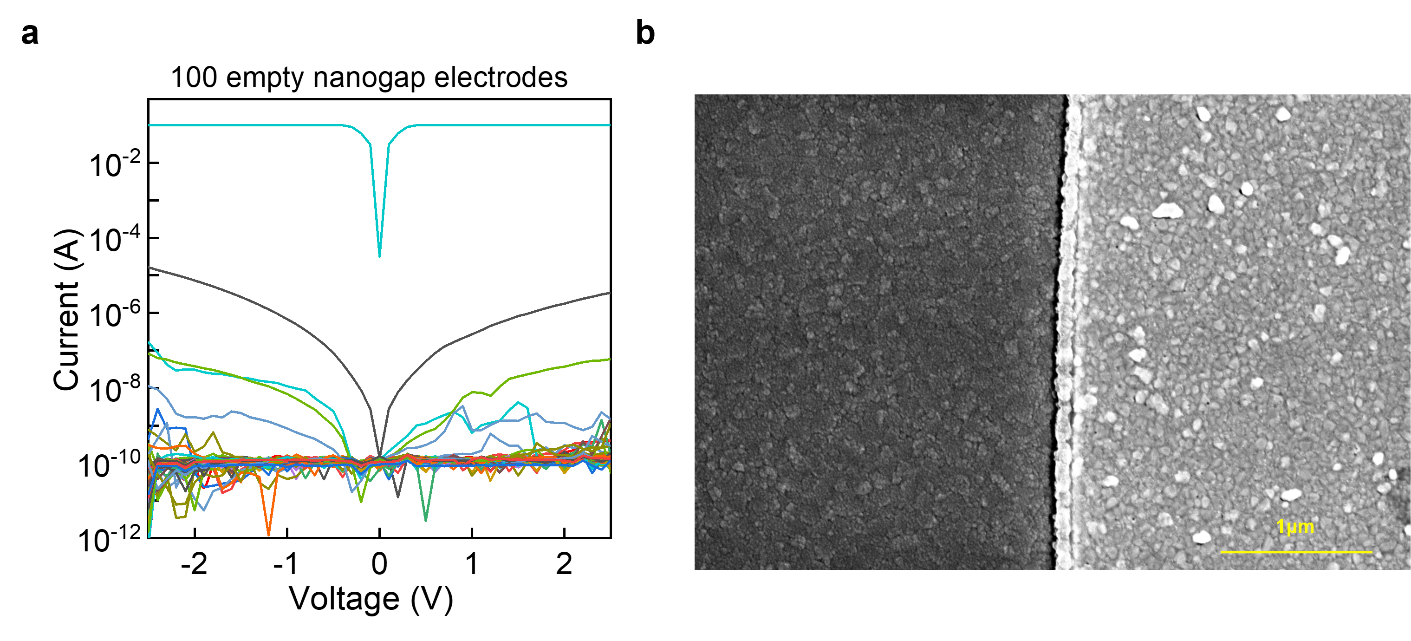


**Figure S18**: a) Electrical measurements of over 100 empty coplanar nanogap electrodes (fabricated without IGZO) to evaluate structural isolation and process reliability. b) SEM image of a representative nanogap region acquired at a large area showing consistent gap formation along the Al/ZnO and Au interface

## **Tables**

**Table S1:** Thermal properties of the materials used in the opto-thermal calculations.

| **Material** | **mass density [g/cm^3^]** | **heat capacity [J/g*K]** | **thermal conductivity [W/m-K]** |
| --- | --- | --- | --- |
| Aluminium | 2.7 | 0.9 | 210 |
| Zinc Oxide | 5.675 | 0.494 | 23.4 |
| Gold | 19.3 | 0.128 | 301 |
| Borofloat Glass | 2.23 | 0.83 | 1.12 |

**Table S2:** IGZO diodes’ junction characteristics extracted from C-V and I-V experimental data.

| **Φ_Β_ (eV)** | | | **Series resistance R_S_ (kΩ)** | | **Richardson constant** | **N_D_** |
| --- | --- | --- | --- | --- | --- | --- |
| **C-V** | **Cheung analysis** | **n** | ***d*V/*d*(ln(I)) vs I** | **H(I) vs I** | **A*(A cm^-2^ K^-2^)** | **(cm^-3^)** |
| 0.58 | 0.44 | 5.2 | 3 | 3.13 | 41 | 1.87 x 10^18^ |

**Table S3:** IGZO diodes’ junction high frequency characteristics.

| **Schottky diode** | **Diode Circumference (mm)** | **f_c,int_ (GHz)**  **(from S11 measurements)** | **f_c,ext_ (GHz)**  **(from V_OUT_ measurements)** | **R (Ω) (from Z(Re) at f_c, int_)** | **C (pF) (from Z(Im) at f_c, int_)** |
| --- | --- | --- | --- | --- | --- |
| **Al/ZnO-IGZO_Au** | 3 | 21 | 5 | 5 | **0.58** |
|  | 0.95 | 131 (simulated) | >110 (linearly extrapolated) | 4.1 | 0.13 |
|  | 0.63 | 184.6 (simulated) | >160 (linearly extrapolated) | 2.9 | 0.08 |

## **References**

[1] J. Semple, D. G. Georgiadou, G. Wyatt-Moon, M. Yoon, A. Seitkhan, E. Yengel, S. Rossbauer, F. Bottacchi, M. A. McLachlan, D. D. C. Bradley, T. D. Anthopoulos, *npj Flex Electron* **2018**, *2*, 18.

[2] G. Wyatt-Moon, D. G. Georgiadou, J. Semple, T. D. Anthopoulos, *ACS Appl. Mater. Interfaces* **2017**, *9*, 41965.

[3] J. Semple, S. Rossbauer, T. D. Anthopoulos, *ACS Appl. Mater. Interfaces* **2016**, *8*, 23167.

[4] G. Wyatt-Moon, D. G. Georgiadou, A. Zoladek-Lemanczyk, F. A. Castro, T. D. Anthopoulos, *J. Phys. Mater.* **2018**, *1*, 01LT01.

[5] D. G. Georgiadou, J. Semple, A. A. Sagade, H. Forstén, P. Rantakari, Y.-H. Lin, F. Alkhalil, A. Seitkhan, K. Loganathan, H. Faber, T. D. Anthopoulos, *Nat Electron* **2020**, *3*, 718.

[6] K. Loganathan, H. Faber, E. Yengel, A. Seitkhan, A. Bakytbekov, E. Yarali, B. Adilbekova, A. AlBatati, Y. Lin, Z. Felemban, S. Yang, W. Li, D. G. Georgiadou, A. Shamim, E. Lidorikis, T. D. Anthopoulos, *Nat Commun* **2022**, *13*, 3260.

[7] S. Kano, T. Kawazu, A. Yamazaki, M. Fujii, *Nanotechnology* **2019**, *30*, 285303.

[8] G. Kresse, D. Joubert, *Phys. Rev. B* **1999**, *59*, 1758.

[9] J. P. Perdew, K. Burke, M. Ernzerhof, *Phys. Rev. Lett.* **1996**, *77*, 3865.

[10] S. Grimme, J. Antony, S. Ehrlich, H. Krieg, *The Journal of Chemical Physics* **2010**, *132*, 154104.

[11] L. Bengtsson, *Phys. Rev. B* **1999**, *59*, 12301.

[12] M. J. Frisch, G. W. Trucks, H. B. Schlegel, G. E. Scuseria, M. A. Robb, J. R. Cheeseman, G. Scalmani, V. Barone, G. A. Petersson, H. Nakatsuji, X. Li, M. Caricato, A. Marenich, J. Bloino, B. G. Janesko, R. Gomperts, B. Mennucci, H. P. Hratchian, J. V. Ortiz, A. F. Izmaylov, J. L. Sonnenberg, D. Williams-Young, F. Ding, F. Lipparini, F. Egidi, J. Goings, B. Peng, A. Petrone, T. Henderson, D. Ranasinghe, V. G. Zakrzewski, J. Gao, N. Rega, G. Zheng, W. Liang, M. Hada, M. Ehara, K. Toyota, R. Fukuda, J. Hasegawa, M. Ishida, T. Nakajima, Y. Honda, O. Kitao, H. Nakai, T. Vreven, K. Throssell, J. A. Montgomery, Jr, J. E. Peralta, F. Ogliaro, M. Bearpark, J. J. Heyd, E. Brothers, K. N. Kudin, V. N. Staroverov, T. Keith, R. Kobayashi, J. Normand, K. Raghavachari, A. Rendell, J. C. Burant, S. S. Iyengar, J. Tomasi, M. Cossi, J. M. Millam, M. Klene, C. Adamo, R. Cammi, J. W. Ochterski, R. L. Martin, K. Morokuma, O. Farkas, J. B. Foresman, D. J. Fox, **2016**.

[13] R. Dennington, T. A. Keith, J. M. Millam, **2016**.

[14] K. Nakamura, T. Takahashi, T. Hosomi, Y. Yamaguchi, W. Tanaka, J. Liu, M. Kanai, K. Nagashima, T. Yanagida, *ACS Omega* **2022**, *7*, 1462.

[15] L. Martínez, R. Andrade, E. G. Birgin, J. M. Martínez, *J Comput Chem* **2009**, *30*, 2157.

[16] “SOPRA database website,” can be found under http://www.sspectra.com/sopra.html, **n.d.**

[17] P. Markoš, C. M. Soukoulis, *Wave Propagation*, Princeton University Press, **2008**.

[18] “Matweb website,” can be found under http://www.matweb.com/, **n.d.**

[19] E. Donchev, P. M. Gammon, J. S. Pang, P. K. Petrov, N. McN. Alford, in (Eds.: L. A. Eldada, M. J. Heben), San Diego, California, United States, **2014**, p. 91770C.

[20] P. Periasamy, H. L. Guthrey, A. I. Abdulagatov, P. F. Ndione, J. J. Berry, D. S. Ginley, S. M. George, P. A. Parilla, R. P. O’Hayre, *Advanced Materials* **2013**, *25*, 1301.

[21] J. Zhang, H. Wang, J. Wilson, X. Ma, J. Jin, A. Song, *IEEE Electron Device Lett.* **2016**, *37*, 389.

[22] S. K. Cheung, N. W. Cheung, *Applied Physics Letters* **1986**, *49*, 85.

[23] D. M. Pozar, *Microwave Engineering*, John Wiley & Sons, Inc, Hoboken, NJ, **2012**.
